# Smartphone App to Address Loneliness Among College Students: Pilot Randomized Controlled Trial

**DOI:** 10.2196/21496

**Published:** 2020-10-20

**Authors:** Emma Bruehlman-Senecal, Cayce J Hook, Jennifer H Pfeifer, Caroline FitzGerald, Brittany Davis, Kevin L Delucchi, Jana Haritatos, Danielle E Ramo

**Affiliations:** 1 Hopelab San Francisco, CA United States; 2 Department of Psychology University of Oregon Eugene, OR United States; 3 Department of Psychiatry and Weill Institute for Neurosciences University of California, San Francisco San Francisco, CA United States

**Keywords:** loneliness, mental health, smartphone app, college, app, student, young adult, randomized controlled trial, efficacy, feasibility, desirability

## Abstract

**Background:**

Loneliness is a widespread and significant problem on college campuses. Prolonged loneliness in young adulthood is a risk factor for concurrent and future mental health problems and attrition, making college a critical time for support. Cognitive and behavioral interventions show promise for decreasing loneliness and can be widely disseminated through technology.

**Objective:**

This pilot randomized controlled trial was conducted to examine the initial efficacy, feasibility, and desirability of a smartphone app, Nod, designed to deliver cognitive and behavioral skill-building exercises to reduce loneliness during the transition to college.

**Methods:**

First-year college students (N=221, mean age 18.7 years, 59% female) were recruited online during incoming student orientation, and randomized to either receive immediate access to Nod (experimental group, n=100) or access after 4 weeks (control group, n=121). The app delivered skills via fully automated (1) “social challenges,” suggested activities designed to build social connections; (2) reflections, brief cognitive reframing exercises; and (3) student testimonials that encouraged a growth mindset toward social connection building. Main intention-to-treat analyses were used to compare the conditions on self-assessed loneliness, depressive symptoms, and other mental health and college adjustment outcomes at week 4, controlling for baseline values on those variables. Analyses were also performed to test the hypothesis that the treatment benefits would be particularly pronounced for participants with heightened psychological vulnerability at baseline (ie, higher baseline depressive symptoms and loneliness).

**Results:**

Retention was 97% at week 4, and participants viewed an average 36.7 pages of app content. There were no significant condition differences in loneliness at week 4 (*F*_1, 211_=0.05, *P=*.82; η_p_^2^ <.001). However, there was a significant condition-by-baseline depression interaction to predict week-4 loneliness (*F*_1,209_=9.65, *P=*.002; η_p_^2^ =.04). Simple slope analyses indicated that baseline depression positively predicted week-4 loneliness among control participants (*r*=0.30, t_209_=3.81, *P*<.001), but not among experimental participants (*r*=–0.09, t_209_=–0.84, *P*=.40), suggesting that Nod buffered participants with high baseline depression scores from experiencing heightened midquarter loneliness. Similarly, there were no significant condition differences in other week-4 outcomes. However, moderation by baseline vulnerability was found for week-4 depressive symptoms, sleep quality, and indices of college adjustment (eg, perceived social support and campus belonging).

**Conclusions:**

Although Nod exposure did not impact outcomes for the full sample, these results provide initial evidence of its benefit for vulnerable students. The results of this trial suggest that cognitive and behavioral skills delivered via a mobile app can buffer psychologically vulnerable college students against heightened loneliness and depressive symptoms, as well as other negative college adjustment outcomes. Future work will aim to improve upon app engagement, and to address loneliness among other key populations.

**Trial Registration:**

ClinicalTrials.gov NCT04164654; https://clinicaltrials.gov/ct2/show/NCT04164654

## Introduction

Loneliness is a painful feeling that arises when there is a discrepancy between one’s desired and achieved patterns of social interactions [1]. Lonely feelings function as an “alarm bell,” signaling that one’s fundamental need for connection and belonging is not being adequately met [2]. Although most of the literature on loneliness focuses on older adults, multiple studies indicate that loneliness is especially prevalent among younger generations [3-5]. For example, in recent national surveys, Generation Z (aged 18-22 years) reported higher loneliness than any other generation surveyed in the United States [6], and 26% of teens and young adults reported they can “never” or “rarely” find companionship when they need it [7]. Loneliness is not just an unpleasant feeling. Prolonged feelings of loneliness in young adulthood are concurrently and prospectively associated with a variety of negative mental health outcomes, including depression, anxiety, social anxiety, and suicidality [8-12]. Compounding these relationships, loneliness is associated with poorer sleep quality [13-15], which can contribute to poorer emotion regulation [16,17] along with further social withdrawal and loneliness in a self-reinforcing cycle [18].

Loneliness in the college context is pervasive and of particular concern: in a 2019 survey, 30% of US undergraduates reported feeling “very lonely” in the last 2 weeks and 67% reported feeling “very lonely” in the last year [19]. Loneliness among college students is associated with lower social adjustment to college [20,21], lower perceived social support [22], and lower campus belonging [23]. In addition to poorer mental health and poorer sleep, college students experiencing heightened loneliness report a greater likelihood of leaving before degree completion [24], as well as lower confidence in their employment prospects and ability to succeed in life [11].

Incoming college students face a major social transition and may be particularly vulnerable to loneliness. Developmental and social psychological evidence indicates that interventions delivered at key moments of transition (such as the transition to college) can substantially impact young adults’ social, academic, and health trajectories [25,26]. As students enter college, they begin to form new routines, new habits, and new relationships that can have powerful recursive effects over time [27]. The college transition thus represents a unique opportunity for intervening to reduce loneliness and improve students’ mental health and academic outcomes.

Loneliness is associated with cognitive biases, including vigilance to social threat and perceptions that others are judging and rejecting [28,29]. Meta-analytic research indicates that the most effective loneliness interventions are those grounded in cognitive behavioral therapy, which target maladaptive cognitions and behaviors [30]. However, most of these interventions have been aimed at older adults, and those designed for college students tend to be resource-intensive. For example, McWhorter and Horan [31] developed an intervention focused on modifying attributional styles with modeling, role playing, and assignments for developing communication skills. The intervention, consisting of six 2-hour structured group experiences led by trained facilitators, significantly decreased participants’ loneliness. Although group-based interventions can effectively reduce loneliness, such interventions may have relatively limited reach in university contexts, where counseling centers are often stretched beyond capacity [32-34]. There is thus a need to develop and test interventions to address youth loneliness at scale.

Mobile apps offer the ability to deliver mental health resources and interventions in a standardized, scalable, and cost-effective manner [35-37]. Smartphone ownership is nearly ubiquitous among young adults [38], and surveys suggest that nearly 1 in 4 smartphone owners aged 18-29 use apps to track or manage health [39]. Further, college counseling centers are increasingly interested in using mobile health apps to disseminate information and interventions to students [40]. Apps provide support on-demand, lowering barriers to much-needed support, such as limited availability of in-person counseling and stigma that can hold back students from seeking help [40]. Prior research has validated the feasibility and acceptability of smartphone app–based loneliness interventions for young people [41,42]. Moreover, systematic reviews of prior psychosocial interventions for youth suggest that technology-based interventions are an appropriate and effective delivery modality for reducing loneliness [43]. However, research is needed to evaluate the efficacy of digital interventions targeting loneliness in undergraduate populations.

We here present the results of a pilot randomized controlled trial of the Nod digital intervention for loneliness among first-year college students (N=221) delivered via a smartphone app. Nod was selected as the intervention in this study because, to our knowledge, it is the only existing mobile intervention specifically designed to address the psychological and behavioral underpinnings of loneliness during the transition to college. Outcomes were compared across two randomly assigned conditions: an experimental group who received 4 weeks of Nod exposure and a waitlist control group given access to Nod after 4 weeks. Our primary hypothesis was that students in the experimental group would report lower loneliness by the end of treatment (week 4) as compared to students in the control group. Secondary hypotheses were that the experimental group would report better outcomes on key mental health indicators associated with loneliness: depression symptoms, anxiety symptoms, social anxiety symptoms, and sleep quality. Exploratory analyses were used to examine effects related to friendship and belonging at the university, namely perceived social support, campus belonging, social adjustment to college, and intention to remain enrolled. Finally, we tested the hypothesis that the treatment benefits would be particularly pronounced for students with heightened psychological vulnerability at baseline, given prior research indicating that targeted interventions have greater effect sizes than universal interventions [44]. Since this was a pilot trial, we also examined app engagement and user experience.

## Methods

### Study Design

This 4-week pilot randomized controlled trial evaluated the initial efficacy, feasibility, and desirability of Nod. At 4 weeks, control participants were given full app access. An 8-week follow-up survey allowed for validation of the main outcome analyses in the control group and an exploration of whether uptake of Nod was similar when delivered later in the school year.

### Participants and Recruitment

Participants were incoming first-year students at a large public university in the northwestern United States, and all recruitment and study procedures were approved by the university Institutional Review Board. Students were eligible for inclusion if they were: (a) entering their first year of undergraduate education, (b) aged 18 to 25 years, (c) English-literate, and (d) not residing with parents/guardians. Students also needed to have a smartphone with an operating system capable of supporting Nod (ie, Mac iOS 9-12 or Android OS 8-10), which 97.9% (806/823) of students who met the four eligibility criteria had.

**Figure 1:**
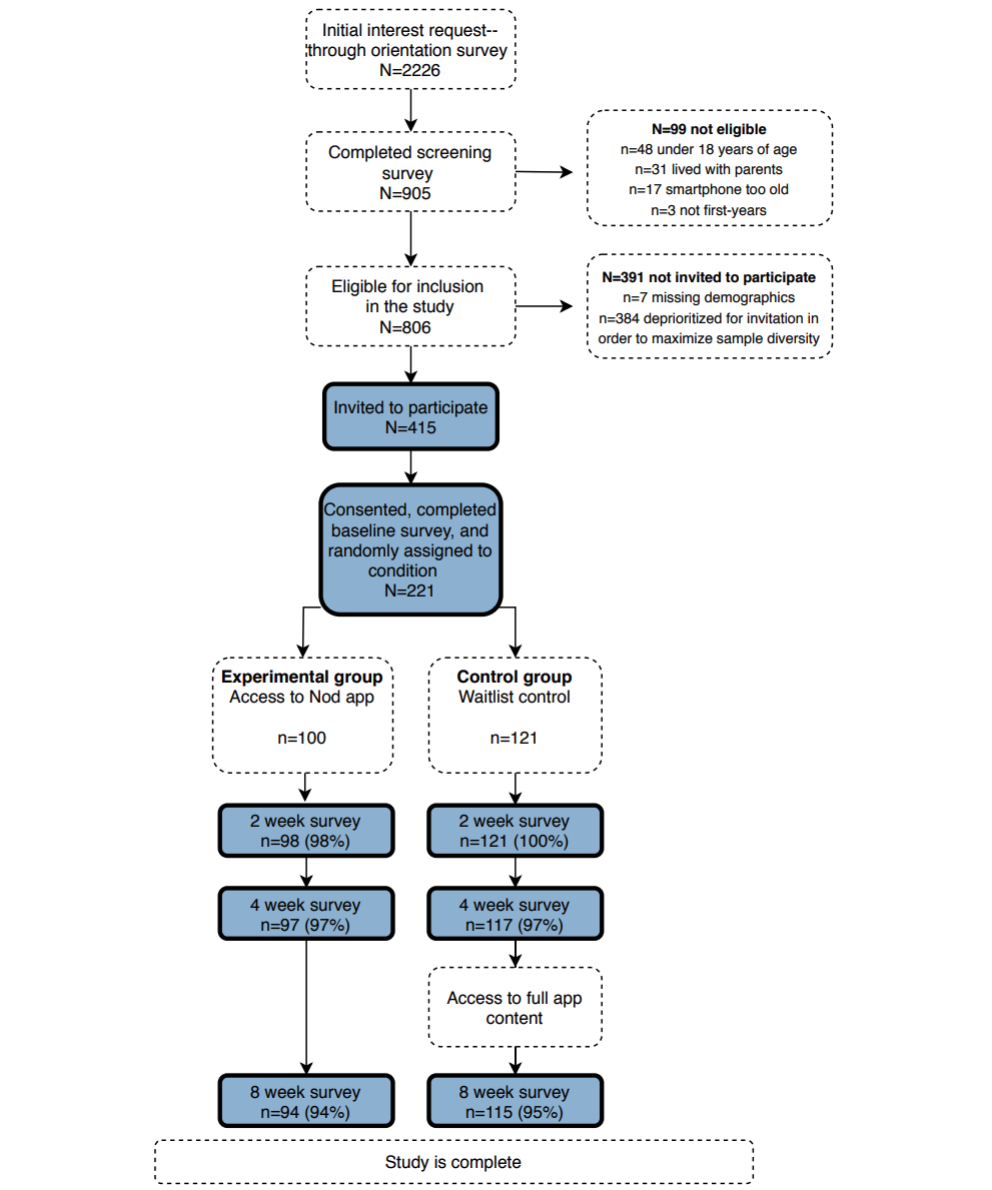
Participant recruitment and flow through the Nod pilot trial.

Participants were recruited from July to September of 2019, in collaboration with the university’s first-year orientation program. All incoming students indicated whether they would like to receive information about a study examining the college transition via a question embedded within a longer orientation survey. Interested participants (N=2226) were sent additional information, and linked to a brief online screening survey containing questions to assess eligibility as well as an 8-item version of the UCLA loneliness questionnaire (UCLA-8) [45]. Of the 905 students who completed the screening survey, 806 met all inclusion criteria (Figure 1). Among these 806 students, we excluded 7 students who did not complete all of the demographic screening questions. The remaining 799 students were divided into those experiencing high loneliness (ie, scoring ≥1 SD above the sample mean on the UCLA-8 loneliness scale, n=176) and those not experiencing high loneliness (n=623). Students in the former group were overrecruited such that they comprised approximately 50% of the sample. Ethnic and racial minority students were also invited to participate at higher rates to achieve a diverse participant pool. Within each loneliness category (high/not high), interested participants were grouped into gender and ethnicity categories, and groups of potential participants were invited to maximize diversity across the sample. In the high-loneliness group, male gender was prioritized due to underrepresentation in the interest pool; in both groups, racial/ethnic and gender minority status were prioritized for similar reasons.

A total of 415 students were invited to participate. This number was selected to ensure that the target enrollment of 220 would be reached with the expectation that not all participants who initially expressed interest in participating would respond to further outreach. Spots in the study were filled on a first-come, first-served rolling basis until the target enrollment was achieved. The enrollment process took 6 days over the second week of classes, and was completed before the third week of classes began. The target sample size of 220 was selected to allow for the detection of condition differences in week-4 outcomes that were medium or larger in size after accounting for potential loss of up to a third of participants due to attrition or noncompliance [46]. In total, 221 participants completed a baseline assessment and were randomized to a study condition. The target enrollment number of 220 was exceeded by 1 participant because 2 control participants accessed the baseline survey at the same time, and thus both were permitted to complete the assessment before automatic survey closure.

### Study Procedure

Informed consent was obtained online, with assessment of understanding used in previous online research [47], immediately prior to completion of the baseline assessment (Multimedia Appendix 1). Following the baseline assessment, participants were randomized 1:1.2 via Qualtrics to either (1) immediate access to Nod (experimental group) or (2) access to Nod following a 4-week waiting period (control). Randomization was stratified after dividing students into two groups: higher loneliness (ie, mean score≥21 on the UCLA-8 screening survey, translating to a loneliness score≥1 SD above the mean of all eligible participants) and lower loneliness (mean score<21 on UCLA-8). Twenty more students were recruited into the control group to account for the possibility that control participants could access Nod prematurely. In-app data confirmed that no control participants did so; therefore, all participants were included in reported analyses. Authors were not blind to participants’ condition during data collection or analysis; however, because randomization was carried out via Qualtrics and all outcome measures were self-assessed by participants, there was no interaction between study staff and participants that could have led to response biases on the part of participants due to demand characteristics.

Within 72 hours of completing the baseline survey, participants in the experimental group were emailed an invitation to download Nod. Those in the control group were notified via email that they would receive access to the app after 4 weeks and advised to await a download invitation. Participants accessed Nod through their university single sign-on, thus preventing an individual from making multiple accounts.

Online assessments through Qualtrics Software (Salt Lake City, UT, USA) were administered at baseline, and at weeks 2, 4, and 8. Participants received US $20 gift cards for (a) creating a Nod account within 1 week of receiving the email invitation, (b) completing each of 4 surveys ($20/survey), and (c) completing all 4 surveys (a $20 bonus), for a possible total of US $120 for their participation. Participants were not incentivized for app usage beyond account registration, and were instructed to use Nod as much or as little as they desired.

### Intervention Conditions

#### Experimental

Nod is a mobile app that was co-developed by Grit Digital Health and Hopelab. Nod incorporates positive psychology, mindfulness-based self-compassion, and cognitive behavioral skill-building exercises to address loneliness among first-year college students. The app delivers skills via three key features: (1) social challenges, suggested ideas for reaching out to others and taking action to build social connections; (2) reflections, short in-app exercises that help students process social experiences and reduce self-criticism; and (3) written student testimonials that encourage a growth mindset toward social connection building. These features were based on exercises and interventions demonstrated to build social connectedness and address negative self and social cognitions in prior empirical research, as described below.

Social challenge content focused on 6 core social skills and behaviors known to strengthen social connections: (1) performing acts of kindness [48,49], (2) expressing gratitude [50,51], (3) active listening [52,53], (4) initiating social outreach/invitations [54,55], (5) being receptive to others’ invitations, and (6) engaging in appropriate self-disclosure [56,57]. Social challenges were designed to encourage in-person socialization within the campus community. Some examples include “Get someone a snack from the dining hall,” and “When you get the urge to bail on a conversation, ask a couple more questions than you normally would, and really listen to the answer.” App content was written to be broadly applicable across campuses (ie, no references were made to university-specific locations or events within the app).

Reflections were short in-app exercises designed to scaffold cognitive restructuring of negative social experiences and savoring of positive social experiences. After completing app-based social challenges, participants were directed to use an interactive mood-rating tool to indicate how they felt about their social experience. Positive mood ratings directed participants to exercises designed to amplify and prolong positive emotions, such as savoring [58] and gratitude [50]. Negative ratings directed participants to cognitive reframing exercises such as self-compassion meditations [59,60] and reappraisal [61,62].

To reinforce a growth mindset toward college friendship [63-65], challenges were accompanied by brief written testimonials (ie, short recommendations of specific in-app social challenges written by college students), which were selected to bolster the belief that forming satisfying social connections takes time and effort.

Users were able to opt to receive intermittent push notification messages that encouraged participants to try new challenges and reflections, to set deadlines for completing challenges, and reminders to come back to the app to mark challenges as completed.

Before launching the pilot trial, Hopelab conducted formative work through interviews, focus groups, and surveys of first-year college students. The app content and visual elements were tailored based on student feedback. Screenshots containing example challenge, reflection, and student testimonial content are presented in Multimedia Appendix 2, and a video describing Nod is provided in Multimedia Appendix 3.

#### Control

Control participants completed the baseline, 2- and 4-week surveys, and received full access to Nod at week 4.

### Measures

#### Engagement

Over 4 weeks, analyses examined (1) the cumulative number of app pages the user accessed, a common measure of app engagement that serves to index the extent of accessed content [66]; (2) the total number of social challenges the user marked as completed; and (3) the total number of reflections clicked through. The latter two measures indexed completion of specific cognitive and behavioral skill-building modules.

#### Loneliness

Loneliness was measured using the UCLA-8 scale. This measure is highly correlated with the longer 20-item version, and its reliability and validity have been established within a college student sample [45]. Participants indicated how frequently they experienced lonely feelings (eg, “I feel left out”) on a 4-point (1=never; 4=often) scale, and items were summed to yield a total score; Cronbach α across all surveys was >.84, indicating high reliability. To increase this measure’s sensitivity to pick up intervention-induced changes, participants indicated their feelings over the past 2 weeks rather than “in general.”

#### Mental Health Indicators

##### Anxiety and Depression Symptoms

Symptoms of anxiety and depression were respectively measured using the 7-item Generalized Anxiety Disorder Scale (GAD-7) [67] and the 9-item Patient Health Questionnaire (PHQ-9) [68]. These brief clinical measures have been widely used to screen for generalized anxiety and depression within a diverse range of settings, including among college students [69-71]. Participants rated the frequency of their symptoms over the prior 2 weeks on a 4-point scale (0=not at all; 3=nearly every day). Items were summed to compute total scores for each construct; Cronbach α across all surveys was >.84 for the PHQ-9 and was >.87 for the GAD-7 (see Multimedia Appendix 4 for the risk assessment protocol associated with this measure.)

##### Social Anxiety Symptoms

The 3-item Mini Social Phobia Inventory, a validated provisional screening tool for social anxiety disorder [72,73], was used to measure social anxiety symptoms over the past week (eg, “Fear of embarrassment causes me to avoid doing things or speaking to people”; 0=not at all, 4= extremely). Items were summed to yield a total score; Cronbach α across all surveys was >.78.

##### Sleep Quality

Subjective sleep quality was measured using one item from the Pittsburgh Sleep Quality Index [74]: “During the past 2 weeks, how would you rate your overall sleep quality?” (0=very good; 3=very bad). Participants indicated how they felt over the past 2 weeks rather than the past month to increase this measure’s sensitivity to intervention-induced changes.

#### College Adjustment Indicators

##### Perceived Social Support

A modified version of the 3-item support subscale of the Comprehensive Inventory for Thriving was used to measure perceived social support [72]. Items were modified to refer to support from people at one’s university (eg, “There are people at [university name] who give me support and encouragement”; 1=strongly disagree, 5=strongly agree), and averaged to yield a total score. Cronbach α across all surveys was >.88.

##### Campus Belonging

Campus belonging was measured using two items adapted from the Student Experiences in the Research University Questionnaire [75], a multi-institutional survey focused on undergraduates’ experiences. Participants rated their agreement with two statements: “I feel like I belong at [university name]” and “I’m happy that I chose to enroll at [university name]” (1=strongly disagree; 6=strongly agree). Items were averaged to yield a total score. Cronbach α across all surveys was >.81.

##### Social Adjustment to College

The 20-item Social Adjustment subscale of the Student Adaptation to College Questionnaire was used to measure adjustment to college social life. Prior research demonstrates the validity of this subscale for predicting college retention [76]. Participants responded to items such as “I’m meeting as many people, and making as many friends as I would like to at [university name]” (1=applies very closely to me; 9=does not apply to me at all). Items were averaged to yield a total score, with higher scores indicating better adjustment; Cronbach α across all surveys was >.89.

##### Intention to Return

Participants’ intention to remain enrolled was measured with a single item adapted from the National Survey for Student Engagement [77]: “Do you intend to return to [university name] in the next year?” (1=definitely yes; 5=definitely not). Responses were skewed, with 69.7% (154/221) of participants at baseline and 64.5% (138/214) at week 4 reporting they would “definitely” return, and were therefore dichotomized (1=definitely yes; 0=all other responses).

#### Demographic Measures

We assessed participants’ age, gender, race/ethnicity, parent/guardian education, subjective socioeconomic status, financial stress, sexual orientation, romantic relationship status, employment status, campus living situation, transfer student status, student athlete status, and autism spectrum status at baseline. All demographic variables were measured via participant self-report.

#### User Experience

A 5-item measure was administered to the experimental group at week 4 and to the control group at week 8 to assess the perceived helpfulness and desirability of Nod (eg, “The Nod app gave me sound advice”; 1=strongly disagree, 7=strongly agree). We computed the proportion of participants endorsing each item (ie, responding “somewhat agree” to “strongly agree”).

Additionally, participants in the experimental group were prompted to give open-ended feedback about the Nod app at weeks 2 and 4 (eg, “What do you find most useful about Nod?” “How could Nod be more helpful to you?”).

### Data Analytic Strategy

#### Engagement

We descriptively compared the engagement of the experimental group (weeks 0-4) to that of the control (weeks 4-8) across each group’s first 4 respective weeks of app exposure. As the distributions of the three engagement variables were highly positively skewed, we report median engagement metrics with their IQRs.

#### Loneliness, Mental Health, and College Adjustment Indices

Analyses of all outcome variables were performed using an intention-to-treat approach, which included all available data from participants randomly assigned to the experimental and control groups. We took a two-step approach to these analyses, reflecting our two main lines of inquiry. In step 1, we tested the primary and secondary hypotheses that the experimental group would report lower loneliness, and other indicators of better mental health and college adjustment at the end of treatment (week 4) as compared to the control group. In step 2, we tested the hypothesis that treatment benefits would be more pronounced for participants with heightened psychological vulnerability at baseline.

Step 1 evaluated condition differences in outcomes at the end of treatment (week 4). Because missing data at week 4 was minimal (213/221, 96.4% of the sample provided full data on all outcome variables), we opted for a straightforward analytic approach that compared the means of the experimental and control groups on each outcome at week 4, adjusting for each outcome’s respective baseline value. A separate analysis of covariance was conducted for each outcome, and each model was evaluated on the basis of the statistical significance (*P*<.05) of the condition term (1=experimental; 0=control). Two outcomes, social adjustment to college and perceived social support, were not measured at baseline, because participants had not yet had enough social experiences on campus to meaningfully answer survey questions. Thus, models for these two outcomes omit baseline scores as a covariate.

Step 2 added an interaction term between baseline vulnerability and condition, allowing us to evaluate whether the benefits of Nod were more pronounced for more vulnerable students. The model of loneliness at week 4 included four predictors: condition, baseline loneliness, baseline depression, and a condition-by-baseline depression interaction term to capture baseline vulnerability. In modeling all other outcomes, models included four predictors: condition, baseline loneliness, baseline score on the outcome variable, and a condition-by-baseline loneliness interaction term. We selected depression as the baseline moderator of week-4 loneliness, and loneliness as the baseline moderator of week-4 depression and all other outcomes, given previous research demonstrating a strong bivariate and reciprocal relationship between loneliness and depression [8,12,78], including in first-year college students [78], and a strong relationship at baseline in this study (*r*=0.52). To determine whether Nod differentially benefitted vulnerable participants, each model was evaluated on the basis of the statistical significance (*P*<.05) of the interaction term.

To validate the results, we separately modeled comparisons between outcomes in the control group at week 8 to outcomes in the experimental group at week 4 (Multimedia Appendix 5).

#### Engagement and Improvement in Outcomes

To explore whether greater engagement with Nod was associated with greater improvement in outcomes, we report correlations between our three measures of engagement and within-participant change in each outcome variable from week 0 to 4 within the experimental group. Due to the skewed distribution of the engagement variables, we report nonparametric (ie, Spearman ρ) correlations. Social support and social adjustment to college, which were not measured at baseline, are excluded from these analyses.

#### User Experience

Within the experimental group, the percentage of users endorsing each desirability statement was reported. Open-ended feedback was analyzed by a single coder using a general inductive approach [79]. Core questions guiding the coding included, “What do students like about Nod?” “What do they wish would change?” and “Based on participant feedback, what factors might improve user experience and engagement with the app?” Quotes were selected to exemplify prominent themes. To validate results, we report quantitative comparisons between user experience of the control group at week 8 to the experimental group at week 4 (Multimedia Appendix 5).

## Results

### Retention

A total of 221 participants completed a baseline assessment and were randomized to study condition (n_experimental_=100; n_control_=121). The rate of follow-up survey completion was high at all time points, and did not differ significantly by condition at any time point (all *P* values >.45; Figure 1).

### Participant Characteristics

Demographic information for the final sample is presented in Table 1. The sample was racially, ethnically, and socioeconomically diverse, with an average age of 18.68 years (SD 0.35, range 18.10-19.77).

**Table 1:** Demographic data of participants.

Characteristic		Total sample (N=221)	Experimental (n=100)	Control (n=121)
Age (years), mean (SD)		18.68 (0.35)	18.66 (0.33)	18.69 (0.36)
**Gender,** **n (%)**				
	Male	81 (36.7)	43 (43.0)	38 (31.4)
	Female	131 (59.3)	51 (51.0)	80 (66.1)
	Nonbinary	9 (4.1)	6 (6.0)	3 (2.5)
**Race,** **n** **(%)**				
	White	117 (52.9)	48 (48.0)	69 (57.0)
	Latino	30 (13.6)	13 (13.0)	17 (14.0)
	Asian/Asian American	21 (9.5)	15 (15.0)	6 (5.0)
	Black	8 (3.6)	2 (2.0)	6 (5.0)
	Native American	2 (0.9)	0 (0.0)	2 (1.7)
	Hawaiian or Pacific Islander	2 (0.9)	2 (2.0)	0 (0.0)
	Two or more races/ethnicities	41 (18.6)	20 (20.0)	21 (17.4)
**Parent/guardian education,** **n** **(%)**				
	High school or less	27 (12.2)	12 (12.0)	15 (12.4)
	Some college (not 4-year)	50 (22.6)	25 (25.0)	25 (20.7)
	One has a 4-year degree	30 (13.6)	13 (13.0)	17 (14.0)
	Both have 4-year degrees	51 (23.1)	24 (24.0)	27 (22.3)
	One has a graduate degree	34 (15.4)	13 (13.0)	21 (17.4)
	Both have graduate degrees	29 (13.1)	13 (13.0)	16 (13.2)
**Subjective SES^a^** **,** **n** **(%)**				
	Low income	15 (6.8)	7 (7.0)	8 (6.7)
	Working class	48 (21.8)	27 (27.0)	21 (17.5)
	Middle class	93 (42.3)	41 (41.0)	52 (43.3)
	Upper middle class	61 (27.7)	24 (24.0)	37 (30.7)
	Wealthy	3 (1.4)	1 (1.0)	2 (1.7)
**Financial stress,** **n** **(%)**				
	Never stressful	8 (3.6)	2 (2.0)	6 (5.0)
	Rarely stressful	43 (19.5)	20 (20.0)	23 (19.0)
	Sometimes stressful	90 (40.7)	46 (46.0)	44 (36.4)
	Often stressful	61 (27.6)	25 (25.0)	36 (29.8)
	Always stressful	19 (8.6)	7 (7.0)	12 (9.9)
**Sexual orientation,** **n** **(%)**				
	Heterosexual	146 (66.1)	69 (69.0)	77 (63.6)
	Gay or lesbian	10 (4.5)	3 (3.0)	7 (5.8)
	Bisexual	36 (16.3)	12 (12.0)	24 (19.8)
	Queer	13 (5.9)	5 (5.0)	8 (6.6)
	Questioning	8 (3.6)	6 (6.0)	2 (1.7)
	Other	5 (2.3)	2 (2.0)	3 (2.5)
	Prefer not to respond or missing	3 (1.4)	3 (3.0)	0 (0.0)
**Relationship status**				
	Single	174 (78.7)	80 (80.0)	94 (77.7)
	Dating	7 (3.2)	4 (4.0)	3 (2.5)
	In a relationship	38 (17.2)	16 (16.0)	22 (18.2)
	Married	1 (0.5)	0 (0.0)	1 (0.8)
	Other	1 (0.5)	0 (0.0)	1 (0.8)
Number of hours of weekly paid employment, mean (SD)		4.98 (7.91)	4.30 (7.47)	5.55 (8.24)
Transfer student, n (%)		1 (0.5)	1 (1.0)	0 (0.0)
**Residence,** **n (%)**				
	Campus residence	215 (97.3)	97 (97.0)	118 (97.5)
	Off campus apt	5 (2.3)	3 (3.0)	2 (1.7)
	Other	1 (0.5)	0 (0.0)	1 (0.8)
**Living situation,** **n** **(%)**				
	Dorm (alone)	1 (0.5)	0 (0.0)	1 (0.8)
	Dorm (roommate)	172 (77.8)	80 (80.0)	92 (76.0)
	Dorm suite (roommates)	37 (16.7)	16 (16.0)	21 (17.4)
	Apartment (with students)	4 (1.8)	3 (3.0)	1 (0.8)
	Apartment (with nonstudents)	1 (0.5)	0 (0.0)	1 (0.8)
	Family	1 (0.5)	0 (0.0)	1 (0.8)
	Other	5 (2.3)	1 (1.0)	4 (3.3)
Student athlete, n (%)		7 (3.2)	2 (2.0)	5 (4.1)
Autism spectrum, n (%)		3 (1.4)	1 (1.0)	2 (1.7)

^a^SES: socioeconomic status.

### Engagement

Ninety-six of the 100 participants (96.0%) in the experimental group and 111 of the 121 participants (91.7%) in the control group created a Nod account within 4 weeks of being granted access to the app. As compared to the control, the experimental group demonstrated descriptively higher engagement with Nod during their first 4 weeks of access, although average engagement was low across both groups (Table 2). Participants in the experimental group viewed a mean of 36.69 pages in the app, while those in the control group viewed a mean of 20.85 pages. We note that although there were 102 pages of total content, users were not expected to progress through all pages in the app sequentially but rather to browse challenges and reflections, and engage as they desired.

**Table 2:** Descriptive statistics for engagement with Nod among first-year college students in the 4 weeks following first access to the Nod app (weeks 0-4 for the experimental group and weeks 4-8 for the control group).

Engagement measures	Experimental: weeks 0-4 (n=96)^a^		Control, weeks 4-8 (n=111)^a^	
	Mean (SD)	Median (IQR)	Mean (SD)	Median (IQR)
Cumulative pages of app content accessed	36.69 (38.50)	23.0 (12.0-43.0)	20.85 (12.71)	17 (10.0-27.0)
Total number of challenges marked as completed by the user	0.89 (1.61)	0.0 (0.0-1.0)	0.31 (0.78)	0.0 (0.0-0.0)
Total number of reflections clicked through	1.13 (1.79)	0.0 (0.0-2.0)	0.37 (0.82)	0.0 (0.0-0.0)

^a^Excludes participants who were randomized to a condition but never created a Nod account (n_experimental_=4 and n_control_=10).

### Loneliness

Descriptive examination of means revealed that both groups’ loneliness scores declined slightly from baseline to week 4 (Table 3). Step 1 of the analyses, which examined condition differences in loneliness at week 4 controlling for baseline scores, showed no evidence for an overall effect of treatment on loneliness (*F*_1,211_=0.05, *P=*.82; η_p_^2^=<.001).

**Table 3:** Loneliness, mental health, and college adjustment outcomes at baseline and week 4 among first-year college students (N=214) receiving the Nod intervention (experimental) versus waitlist (control).

Outcome	Baseline^a^, mean (SD)		Week 4, mean (SD)	
	Experimental	Control	Experimental	Control
Loneliness (UCLA-8^b^)	18.87 (4.32)	18.91 (4.40)	16.71 (4.73)	16.87 (5.32)
Depression (PHQ-9^c^)	5.31 (4.18)	6.65 (5.52)	5.71 (4.14)	7.12 (5.90)
Anxiety symptoms (GAD-7^d^)	5.90 (4.31)	6.85 (5.10)	5.22 (4.24)	6.50 (5.39)
Social anxiety symptoms (Mini-SPIN^e^)	5.21 (2.89)	5.25 (3.23)	4.19 (3.20)	4.54 (3.45)
Sleep quality (PSQI^f^)	1.20 (0.62)	1.33 (0.78)	1.21 (0.64)	1.38 (0.77)
Perceived social support (CIT^g^ subscale)	not measured	not measured	4.20 (0.67)	4.08 (0.77)
Campus belonging (SERU^h^)	5.00 (0.89)	4.96 (0.89)	4.94 (1.00)	4.86 (0.99)
Social adjustment to college (SACQ^i^ subscale)	not measured	not measured	6.07 (1.26)	5.92 (1.50)
Intention to return (NSSE^j^)	0.68 (0.47)	0.71 (0.46)	0.69 (0.46)	0.61 (0.49)

^a^Baseline scores exclude data from 7 participants who were missing data at week 4.

^b^UCLA-8: UCLA Loneliness Scale, 8-item.

^c^PHQ-9: Patient Health Questionnaire, 9-item.

^d^GAD-7: Generalized Anxiety Disorder, 7-item scale.

^e^Mini-SPIN: Mini Social Phobia Inventory.

^f^PSQI: Pittsburgh Sleep Quality Index (higher scores reflect lower quality sleep).

^g^CIT: Comprehensive Inventory of Thriving.

^h^SERU: Student Experiences in the Research University Questionnaire.

^i^SACQ: Student Adaptation to College Questionnaire.

^j^NSSE: National Survey for Student Engagement.

**Figure 2:**
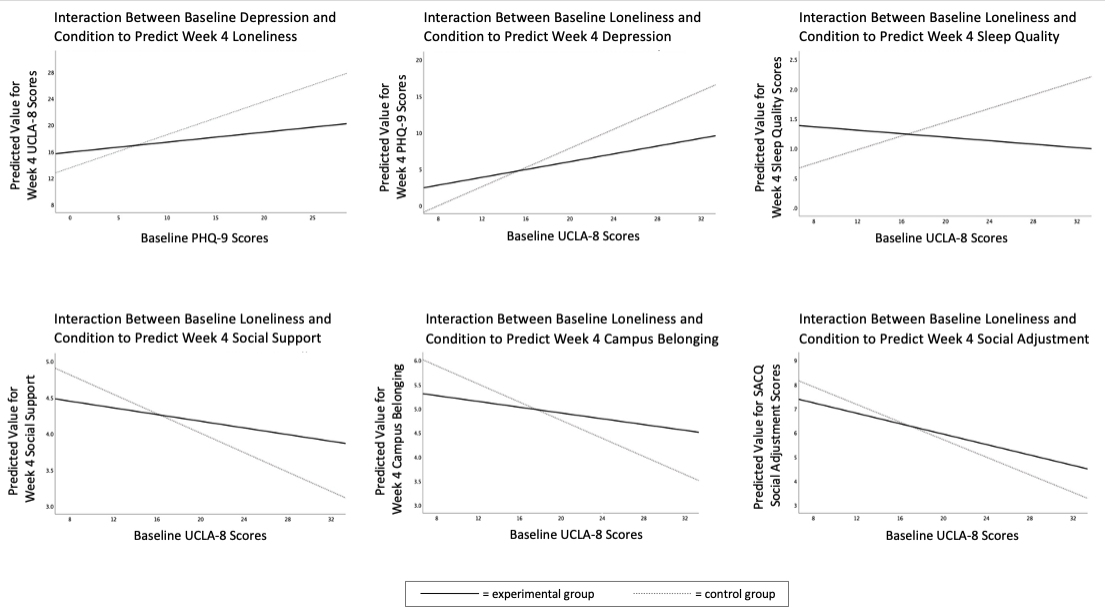
Simple slopes of baseline vulnerability on select week-4 mental health and college adjustment outcomes in the experimental vs control groups. All graphs represent complete case analyses. Higher sleep quality scores indicate lower quality sleep. UCLA-8: UCLA Loneliness Scale, 8-item; PHQ-9: Patient Health Questionnaire 9-item; SACQ: Student Adaptation to College Questionnaire.

Step 2 of the analyses revealed a significant condition-by-baseline depression interaction (*F*_1,209_=9.65, *P=*.002; η_p_^2^=.04). To interpret this interaction, we conducted follow-up analyses of the simple slopes of baseline depression on week-4 loneliness for each condition separately. Within the control group, there was a significant positive relationship between baseline depression and week-4 loneliness. In contrast, there was no significant relationship between baseline depression and week-4 loneliness within the experimental group (Table 4), suggesting that Nod buffered participants high in baseline depression from experiencing heightened midquarter loneliness (Figure 2).

**Table 4:** Simple slopes for loneliness, mental health, and college adjustment outcomes at week 4 among first-year college students receiving the Nod intervention (experimental) versus waitlist (control).

Outcome			*r* of simple slope^a^		*t* value		df		*P* value
**Loneliness (UCLA-8^b^** **)**									
	Experimental	–0.09		–0.84		209		.40	
	Control	0.30		3.81		209		<.001	
**Depression (PHQ-9^c^** **)**									
	Experimental	–0.04		–0.44		209		.66	
	Control	0.23		2.60		209		.01	
**Sleep quality (PSQI^d^** **)**									
	Experimental	–0.02		–1.24		208		.22	
	Control	0.04		2.89		208		.004	
**Social support (CIT^e^** **subscale)**									
	Experimental	–0.02		–1.41		209		.16	
	Control	–0.07		–4.60		209		<.001	
**Campus belonging (from SERU^f^** **)**									
	Experimental	0.03		1.54		209		.13	
	Control	–0.05		–2.70		209		.007	
**Social Adjustment to College (SACQ^g^** **subscale)**									
	Experimental	–0.11		–3.73		209		<.001	
	Control	–0.18		–7.05		209		<.001	
**Intention to return to college (NSSE^h^** **)**									
	Experimental	1.15 (1.01-1.32)		2.16		209		.03	
	Control	0.89 (0.80-1.00)		–2.01		209		.05	

^a^Except for intention to return to college, which was assessed based on odds ratio (95% CI).

^b^UCLA-8: UCLA Loneliness Scale, 8-item.

^c^PHQ-9: Patient Health Questionnaire 9-item.

^d^PSQI: Pittsburgh Sleep Quality Index (higher values indicate poorer quality sleep).

^e^CIT: Comprehensive Inventory of Thriving.

^f^SERU: Student Experiences in the Research University Questionnaire.

^g^SACQ: Student Adaptation to College Questionnaire.

^h^NSSE: National Survey for Student Engagement (1=will definitely return; 0=all other responses).

### Mental Health Indicators

Analyses in step 1 showed no evidence for an overall effect of treatment on any of the four indices of mental health (ie, week-4 depression, anxiety, social anxiety, or sleep quality): all *F* values were <1.60 and all *P* values were >.20. Step 2 of the analyses revealed a significant condition-by-baseline loneliness interaction to predict week-4 depression (*F*_1,209_=5.17, *P=*.02, η_p_^2^=.02) and week-4 sleep quality (*F*_1,208_=8.26, *P=*.004, η_p_^2^=.04). Similar to the pattern observed for week-4 loneliness, simple slope analyses indicated that Nod buffered participants with higher baseline loneliness against heightened midquarter depression and poor sleep quality (Table 4; Figure 2). Baseline loneliness did not significantly moderate the effect of condition on week-4 anxiety or social anxiety (both *F* values<1.80, *P*>.18).

### College Adjustment Indicators

There was no evidence for an overall effect of treatment on any of the three indices of college adjustment (ie, week-4 social support, campus belonging, or social adjustment to college); all *F* values were <1.40 and all *P* values were >.23. However, the experimental group was more likely to report that they definitely intended to return to campus in the upcoming school year compared with the control (odds ratio=2.11, 95% CI 1.00-4.49, *z*=1.95, *P*=.05).

Step 2 of the analyses revealed a significant condition-by-baseline loneliness interaction to predict week-4 social support (*F*_1,210_=4.05, *P=*.045; η_p_^2^=.02) and campus belonging (*F*_1,209_=9.44, *P=*.002; η_p_^2^=.04). The condition-by-baseline loneliness interaction to predict week-4 social adjustment to college approached but did not reach statistical significance (*F*_1,210_=3.66, *P=*.06; η_p_^2^=.02). Simple slope analyses suggested that Nod buffered participants with higher baseline loneliness against reduced social support, campus belonging, and social adjustment at week 4 (Table 4 and Figure 2).

Additionally, the significant main effect of condition on intention to return was moderated by a condition-by-baseline loneliness interaction (odds ratio=1.29, 95% CI 1.09-1.54, *z*=2.90, *P*=.004). Probing of this interaction revealed that within the control group, the odds of “definitely” intending to return to campus significantly decreased as baseline loneliness increased. In contrast, in the experimental group, the odds of intending to return significantly *increased* as baseline loneliness increased (Table 4).

### Engagement and Outcome Improvement

Within the experimental group, the three indicators of engagement with Nod from week 0 to 4 were weakly positively associated with outcome improvement across a broad array of mental health and college adjustment indices, including loneliness, although many of these associations failed to reach statistical significance (Table 5).

**Table 5:** Spearman correlation coefficients (ρ) between engagement with Nod and change in outcomes from baseline to week 4 within the experimental group (N=94).^a^

Outcome	Total number of app pages clicked through		Total number of challenges marked as completed		Total number of reflections completed	
	ρ	*P* value	ρ	*P* value	ρ	*P* value
Loneliness (UCLA-8^b^)	–0.20	.06	–0.23	.03	–0.17	.11
Depressive symptoms (PHQ-9^c^)	–0.16	.13	–0.20	.05	–0.02	.88
Anxiety symptoms (GAD-7^d^)	–0.24	.02	–0.26	.01	–0.17	.10
Social anxiety symptoms (Mini-SPIN^e^)	–0.02	.82	–0.02	.85	0.01	.90
Sleep quality (PSQI^f^)	0.07	.51	–0.002	.99	0.06	.59
Campus belonging (from SERU^g^)	0.08	.43	0.07	.52	0.16	.13
Intention to return (from NSSE^h^)	0.13	.21	0.08	.46	0.18	.08

^a^Only participants in the experimental group who created an account within Nod during the first 4 weeks in the study were included in these analyses.

^b^UCLA-8=UCLA Loneliness Scale, 8-item.

^c^PHQ-9=Patient Health Questionnaire 9-item.

^d^GAD-7=Generalized Anxiety Disorder, 7-item scale.

^e^Mini-SPIN=Mini Social Phobia Inventory.

^f^PSQI Sleep Quality=Sleep Quality item from the Pittsburgh Sleep Quality Index (higher values indicate lower quality sleep).

^g^SERU=Student Experiences in the Research University Questionnaire.

^h^NSSE=National Survey for Student Engagement.

### User Experience

The majority of participants in the experimental group rated the app as easy to understand, and agreed that Nod gave them sound advice and something new to think about. However, fewer participants indicated that they would like to continue to use Nod, or had used what they learned in daily life (Table 6).

When asked what they found most useful about Nod, the majority of participants noted that Nod gave them new ideas for socializing or new ways of reflecting on social experiences: “Nod allows me to think of ways to interact with people that I probably wouldn’t have thought of on my own. It opens more opportunities for me.”

Additional benefits related to increased confidence to push outside of one’s social comfort zone, social goal setting, and accountability, and the simple user experience design.

I’ve been more outgoing. The challenges I set up for myself really help me push my comfort zone to socialize more than I usually do.

It’s nice that the app has actual goals for you to do. I try to set social goals for myself, but this app makes me more accountable and really encourages me to be creative in social interaction. It’s actually really fun!

I think the simplicity of the app makes it effective...

When asked what they would change about Nod and how it could be more helpful, a majority of participants expressed a desire for greater personalization. For example, one participant stated: “I would maybe add in the ability to make your own interaction goals and give more of an ability to track your progress.”

Other notable themes included wanting more and different types of push notifications, requesting the addition of social networking features, and suggesting improvements to app gamification.

I would like to be able to establish more connections with people through the app—it can be difficult for me to introduce myself to people face-to-face so having that option might be of use to someone like me.

Give students points or rewards or something that makes students feel like they should take the tips.

Send daily reminders, facts, recommendations, encouragement.

**Table 6:** Proportion of participants in the experimental group who responded “somewhat agree,” “agree,” or “strongly agree” to the respective statements (N=97).

Statement	Respondents in agreement, n (%)
The content of the Nod app was easy to understand.	81 (84)
The Nod app gave me sound advice.	74 (76)
The Nod app gave me something new to think about.	72 (74)
I’d like to continue to use the Nod app.	45 (46)
I’ve used what I’ve learned from Nod in my daily life.	40 (41)

## Discussion

### Principal Results

Intention-to-treat analyses indicated that there were no significant overall effects of the Nod app on loneliness, mental health, or college adjustment outcomes. However, Nod did have significant benefits for students who entered college with elevated risk (ie, heightened loneliness and depression) relative to their peers. Exposure to Nod buffered vulnerable first-year college students from experiencing heightened mid-semester loneliness and depressive symptoms, and protected against poor sleep quality, reduced social support, and reduced campus belonging. Notably, the experimental group was more likely to report that they would definitely return to campus in the upcoming school year, a benefit that was particularly pronounced for vulnerable students who are at heightened risk of early attrition [24]. These results support using app-based interventions to facilitate social connection, especially among first-year students experiencing elevated loneliness or depressive symptoms during key moments of social transition.

Less vulnerable students (ie, those with average to low levels of baseline loneliness and depression) did not derive significant benefits from Nod. These students may have had less need for the provided skills, and thus may have used Nod less frequently and benefited from it less. This possibility is supported by exploratory analyses demonstrating that baseline loneliness was positively associated with all measures of app engagement in the experimental group (all *r*>.27, all *P*<.05), indicating that more vulnerable participants used Nod more frequently than less vulnerable participants. This pattern is consistent with the broader study findings that students higher in baseline vulnerability derived greater benefit from being assigned to use Nod.

However, on average, even vulnerable students did not engage with Nod extensively, raising questions regarding what elements of Nod usage account for its benefits. Prior research has demonstrated that very brief (eg, 1 hour) growth mindset and social belonging interventions, when delivered at key points of social transition, can have prolonged positive effects on student well-being and achievement [27,65,80]. One possibility is that the social growth mindset messaging woven into Nod might have set in motion recursive psychosocial processes that accumulated over time. For example, reading student testimonials that normalize feelings of nervousness or awkwardness, and receiving prompts to try out new social activities, may have encouraged vulnerable students to take small social risks early in college (eg, to strike up new conversations or to go out to an event rather than staying home), which may have in turn set the stage for future patterns of positive interaction without requiring extensive engagement with the app. It is also possible that Nod’s benefits might accrue from the additional socializing that Nod encourages students to engage in “in real life,” regardless of whether students return to the app to mark challenges as complete. The goal of Nod, and indeed many app-based behavior-change interventions, is not engagement with the app per se, but engagement in behaviors that are the target of the intervention—in Nod’s case, supportive social interactions. Future research might seek to measure these real-world engagements to better explore the mechanisms by which Nod supports the well-being of vulnerable students.

Participants’ engagement data and qualitative feedback indicated several strengths of Nod as well as areas for improvement. The majority of participants agreed that the content was easy to understand and that Nod gave them new ideas. However, less than half of the participants indicated that they would like to continue to use Nod after the trial ended, and the majority did not mark any challenges as completed within the app. Several factors may have impeded continued engagement. The majority of participants expressed that Nod would benefit from greater personalization such as challenges that adapt to the user’s comfort level. Participants also indicated that they would benefit from more and different types of push notifications, and suggested providing more in-app incentives. Future work will aim to boost motivation and reminders to engage through increased notifications, gamification, and clearer description of the potential benefits of app usage to students.

### Limitations

Several limitations motivate further investigation. First, on average, participants did not engage extensively with Nod, raising questions regarding what elements of Nod usage account for its benefits to vulnerable students. Future research should incorporate finer-grained measures of in-app feature use, including details on how many and which specific challenge titles, tips, and testimonials students view, as well as out-of-app social behavior, to explore in greater depth the question of how Nod has beneficial effects. Future research should examine additional measures of engagement, including daily session duration, number of sessions per week, trends in use over time, and cumulative time spent in the app, as well as the proportion of students accepting push notifications, and examine possible differences in outcomes across these engagement metrics. Doing so may facilitate a clearer understanding of how Nod supports mental health, as well as the minimum effective exposure needed to achieve positive effects. To improve engagement, developers could also investigate whether additional features such as video testimonials, the ability to connect with and message other users, or the inclusion of a module with links to campus-specific resources such as information on mental health resources, local hangouts, and interest groups might draw more students to return to the app.

A second limitation was the relatively small sample size of this initial pilot trial and its inclusion of a single university. Future larger trials should be conducted on a diverse range of college campuses, including commuter campuses and those with diverse student populations. In this trial, more women than men expressed interest in participating. Future user research aimed at increasing the appeal of study recruitment materials among college-aged men could help to ameliorate this gender imbalance.

Although there is precedent for loneliness interventions significantly reducing loneliness by 4 weeks [81-83], future studies would also benefit from longer follow-up periods to understand the magnitude and longevity of effects over time. Additionally, similar to many other pilot studies of tech-based behavioral interventions, it was not feasible to blind participants to condition. Future studies could deliver a “dummy” (ie, “sham”) app to blind control participants and strengthen evidence for the efficacy of Nod.

Finally, participants were motivated to participate in a trial and were incentivized to download Nod, potentially weakening the generalizability of the findings. A naturalistic study of engagement outside of a clinical trial could provide more generalizable insights regarding Nod’s benefits.

### Comparison With Prior Work

Despite recent research demonstrating that 18-22 year olds report higher loneliness than any other generation, most loneliness interventions have been designed for older adults [84]. Systematic reviews of prior loneliness interventions identified a need for theoretically driven and rigorously evaluated interventions for loneliness in younger populations [43,84]. Our randomized controlled design and use of theory-driven strategies to reduce loneliness in college students helps to fill a significant gap in the research literature. To our knowledge, this study represents the first randomized controlled trial of a scalable, universal mobile intervention to address loneliness during the transition to college—a time of particular vulnerability.

Our finding that the beneficial effects of Nod exposure were most pronounced among vulnerable students accords with prior research. First, previous interventions for loneliness in undergraduate populations specifically recruited students experiencing heightened loneliness or depressive symptoms, as these populations were deemed most in need of resources [31,81]. Second, meta-analytic research suggests that mental health interventions have larger effects when targeting vulnerable populations [44]. Nevertheless, Nod’s intentional design to be appropriate for a universal audience offers several benefits. By avoiding identifying students as “in need” of targeted support, Nod avoids stigmatizing users. Nod can also be delivered before students present with problems at counseling centers, allowing upstream prevention without requiring screening of at-risk students.

### Conclusions

This study demonstrated that a smartphone app can provide scalable, self-paced, and confidential support for students to prevent and cope with loneliness. Exposure to Nod buffered against heightened loneliness and depression, and resulted in enhanced sleep quality, campus belonging, social support, and intention to return to college among vulnerable first-year students. Given its simple user interface and a format that supports iteration on content, the app is likely to appeal to a broad range of students. The randomized design of this trial extends the promising findings of similar interventions for college students [41], and bolsters confidence that loneliness can be addressed digitally. Future work will aim to improve upon app engagement, and to address loneliness during other key social transitions and among other young populations who may benefit from digital interventions to support social connectedness. 

## Appendix

Multimedia Appendix 1 Consent form.

Multimedia Appendix 2 Nod challenge, testimonial, and reflection screenshots.

Multimedia Appendix 3 Nod informational video.

Multimedia Appendix 4 Nod risk assessment protocol.

Multimedia Appendix 5 Comparisons between outcomes and engagement in the control group at week 8 to the experimental group at week 4.

Multimedia Appendix 6 CONSORT-eHEALTH checklist (v1.6.1).

## Abbreviations

GAD-7: 7-item Generalized Anxiety Disorder Scale
PHQ-9: 9-item Patient Health Questionnaire
UCLA-8: 8-item version of the UCLA loneliness questionnaire
