# Supplementary material for: Smartphone App to Address Loneliness Among College Students: Pilot Randomized Controlled Trial
Source: JMIR Ment Health. 2020 Oct 20;7(10):e21496. doi: 10.2196/21496 (PMC7609198; doi:10.2196/21496)
Supplement: Multimedia Appendix 1 [file mental_v7i10e21496_app1.pdf]

Appendix 1. Nod Consent Form

Thanks for your interest in The Freshman Project 3! We will now show you our consent form. At the end of this consent form are the following questions which you will need to answer:

1. What is this study about?
2. How long am I required to be in this study?
3. What are some of the possible risks associated with participating in this study?

These questions will help us assess your understanding of the study, your rights as a participant, and the possible risks associated with your participation.

Appendix 1. Nod Consent Form

CONSENT TO PARTICIPATE IN AN ONLINE STUDY

You are being asked to participate in a research study conducted by Dr. Jennifer Pfeifer's Developmental Social Neuroscience laboratory in the Department of Psychology at the University of Oregon and Dr. Danielle Ramo from Hopelab. Hopelab is a nonprofit foundation based out of San Francisco that works to improve the health and wellbeing of teens and young adults. The box below highlights key information about this research for you to consider when making a decision whether or not to participate. Carefully consider this information and the more detailed information provided below the box. Please ask questions about any of the information you do not understand before you decide whether to participate.

Key Information for You to Consider
• **Voluntary Consent**. You are being asked to volunteer for a research study. It is up to you whether you choose to participate or not. There will be no penalty or loss of benefits to which you are otherwise entitled if you choose not to participate or discontinue participation.<br>• **Purpose**. The purpose of this research is to examine the effects of using a mobile app on social processes and well-being during the transition to college. By being in this study, you will help us learn more about the social transition to college and whether an app can help with this transition.<br>• **Duration.** It is expected that your participation will last eight weeks.<br>• **Procedures and Activities**. You will be randomly assigned to either receive the app right away or receive the app after a four-week waiting period. You will also be asked to complete four online surveys, which will ask you questions about your college experience and psychological wellbeing. Each survey will take 25-30 minutes to complete. You will be compensated up to $120 for your participation.<br>• **Risks**. Some of the foreseeable risks or discomforts of your participation include psychological discomfort associated with answering survey questions. There is also a risk of loss of privacy/confidentiality, which is described below.<br>• **Benefits**. It is possible that participating in this study might help you to learn new coping skills. In a broader sense, by becoming involved in this project you will be contributing to the advancement of scientific research in the field of psychology.<br>• **Alternatives.** Participation is voluntary and the only alternative is to not participate.

• PURPOSE OF THE STUDY

The study we are working on looks at young adults' health, well-being, and social processes during the transition to college. Specifically, it examines the effects of using a mobile app, on social process and well-being. This app was co-created by Hopelab and Grit Digital Health, and is operated by Grit Digital Health. By being in this study, you will help us learn more about the social transition to college and whether an app can help with this transition.

• PROCEDURES

Step 1. Read this consent form and decide if you want to be part of the study.
Step 2. Once you decide to be part of the study, you will be assigned to 1 of 2 groups. We expect that your participation will last eight weeks.
Group 1 will have immediate access to the app.
Group 2 will receive access to the app after a 4-week waiting period.
During the waiting period both groups will complete study surveys on the schedule

described below. Your group assignment will be given in a message sent immediately upon completion of the first of the four surveys. Participants are put into groups randomly, so you can't choose which group to be put into.
Step 3. You will complete 4 surveys during the study. The surveys will be collected online via Qualtrics. You will complete the first of four surveys after providing informed consent, and you will then receive an email each time a new survey is available. Each survey will take about 25-30 minutes to complete. The surveys will have questions about your psychological well-being and experiences in college. You will also be asked to interact with the app throughout the duration of your participation. The app is designed for engagements that average around 5 minutes in length. You are free to use the app as frequently as you like and find helpful. The amount of time you spend in the app is unrelated to your study compensation.

• POTENTIAL DISCOMFORTS AND RISKS THAT YOU MAY EXPERIENCE AND STEPS WE WILL TAKE TO LESSEN POTENTIAL DISCOMFORTS AND RISKS
It is possible you may feel uncomfortable or experience emotional distress while working with the app or answering the surveys. We will ask questions about your psychological well-being, your emotions, your social experiences, and how you like the app. If you feel uncomfortable at any point you may 1) stop participating at any time without penalty or 2) elect to skip any or all questions that make you uncomfortable. There is also a risk of loss of privacy/confidentiality, for more information read the "Confidentiality" section below.

• ANTICIPATED BENEFITS TO PARTICIPANTS
Although you will be compensated for your time completing the questionnaires, there are no demonstrated benefits to participation. It is possible that participating in this study might help you to learn new coping skills presented through the app. In a broader sense, by becoming involved in this project you will be contributing to the advancement of scientific research in the field of psychology and in the examination of whether the app supports the well-being of college students.

• ALTERNATIVES TO PARTICIPATION
Since this study is not designed to treat participants for anything, the only alternative to participating in the study is not to participate.

• PAYMENT FOR PARTICIPATION
If you decide to participate, you will be compensated $20 per questionnaire and a bonus of $20 for completing all four questionnaires. Additionally, you will also receive a $20 bonus if you create an account within the app within 1 week of receiving an initial email invitation to access the app. Thus the total compensation for this study is up to $120 in total. Compensation will be in the form of Amazon electronic gift cards.

• FINANCIAL OBLIGATION, EMERGENCY CARE, COMPENSATION FOR INJURY
Neither you nor your insurance company will be billed for any costs associated with your participation in this research. We take your safety very seriously and rigorously follow extremely high safety standards. It is, therefore, highly unlikely that you would be injured in the course of this research study, but if you were to be physically injured because of the project, you and/or your insurance company would be responsible for your doctor bills. If you are a University of Oregon student or employee and are covered by a

University of Oregon medical plan, that plan might have terms that apply to your injury.

• PRIVACY AND CONFIDENTIALITY

To better ensure the privacy and confidentiality of your personal information, your data will be anonymized so that you cannot be easily identified. This means we separate information that could personally identify you (e.g. your name, email address, phone number, etc.) from both your survey data and your app usage data. We will do so by assigning a unique ID number to you (e.g.,10001, 10002, 10003, etc.) so that we can tell which data are yours without knowing who you are personally. We will keep your identifying information and its associated code number in a separate locked location or in a password protected computer file on a temporary basis only. Only Dr. Pfeifer and Dr. Ramo will have access to the list of identifiers. No information about your identity will be disclosed to others without your written permission, except if necessary to protect your rights or welfare (for example, if you were to be injured and needed emergency care) or if required by law. Please note, if your answers to survey questions indicate a risk of harm to yourself, confidentiality will be broken, and study staff from Dr. Pfeifer's lab will access your identifying information in order to reach out to you directly by email or phone. When the results of this research are published or discussed in conferences, absolutely no information will be included that would reveal your identity.

Your in-app data will adhere to the privacy and confidentiality rules established by Grit Digital Health. If you have questions or concerns about these confidentiality rules please contact the research team listed above and do not proceed with participating in this study until your questions have been answered. You will be asked to create an account on the app using your DuckID credentials, and we will use your unique ID number to connect your survey answers with other information about you that you report within the app. The app will be structured in a way that all participant identifiable information will be encrypted immediately upon account creation to ensure that no individual from outside the research team will match user behavior or data to an individual participant. After removal of all personally identifiable information, the data you provide in this study could be used for future research studies or distributed to another investigator for future research studies without additional informed consent. However, absolutely no information will be included in this data that could reveal your identity.

At any time, you can choose to stop being in the study by calling or emailing the Principal Investigator listed at the top of this form. If you ask to not continue:

· We won't ask for any new information about you
· We'll use whatever information you already shared

• PARTICIPATION AND WITHDRAWAL

Your participation is voluntary. Your decision whether or not to participate will not affect your relationship with the Psychology Department, the University of Oregon, Dr. Pfeifer, Dr. Ramo, Hopelab, or the Developmental Social Neuroscience Lab. If you decide to participate, you are free to withdraw your consent and discontinue participation at any time without penalty. You may choose not to complete any aspects of the study (e.g., individual questionnaire items, in app interactions) without penalty. However, in the case that you choose not to complete the entire questionnaire, the experimenter may discontinue the study without penalty to you.

• WITHDRAWAL OF PARTICIPATION BY THE RESEARCHER

Circumstances may arise which might cause the researcher to end your participation in sessions before the completion of the study. This decision may be made to protect your

health and safety, or because we determined based on your experiences in past sessions that you are not eligible to continue to take part in one or more future sessions. In this case, the decision will be explained to you.

• NEW FINDINGS
During the course of the study, you will be informed of any significant new findings (either good or bad), such as changes in the risks or benefits resulting from participation in this research that might cause you to change your mind about continuing to participate in this study. If significant new information is provided to you, we will ask for a renewal of your consent, reflecting the new information.

• IDENTIFICATION OF RESEARCH INVESTIGATORS
If you have any questions about the research, or if you experience an injury or negative reaction related to this research, please contact the following research investigators:

Jennifer Pfeifer, Ph.D.
Department of Psychology
1227 University of Oregon
Eugene, OR 97403-1227
Office: (541) 346-1984
jpfeifer@uoregon.edu

Danielle Ramo, Ph.D.
Hopelab
100 California St.
San Francisco, CA 94111
Office: (415) 818-8411
dramo@hopelab.org

• RIGHTS OF RESEARCH PARTICIPANTS
You may withdraw your consent at any time and end participation without penalty. You are not waiving any legal claims, rights or remedies because of your participation in this research study. If you have questions regarding your rights as research participants, you may contact Research Compliance Services, 5237 University of Oregon, Eugene, OR, 97403, (541) 346-2510, ResearchCompliance@uoregon.edu.

Appendix 1. Nod Consent Form

To begin the questionnaire, please click the start button below. By clicking this button, you are indicating that you have read and understand the information provided above, that you willingly agree to participate, that you may withdraw your consent at any time and discontinue participation without penalty. If you would like to keep a copy of this information for your records, please print or save this page.
If you do NOT want to participate, please exit now or direct your browser elsewhere.

Are you ready to answer the questions outlined earlier?

Appendix 1. Nod Consent Form

◯ Yes (1)

◯ I want to review once again (2)

Display This Question:

If Are you ready to answer the questions outlined earlier? = I want to review once again

◯ Click to download a PDF of the consent

◯ When ready click NEXT to move forward.

End of Block: [Consent Form]

Start of Block: [[Consent Questions]]—correct answers highlighted below

1. What is this study about?

◯ My academic performance (1)

◯ The social transition to college (2)

◯ Diet and exercise (3)

2. True or false. If I am found eligible I will HAVE to participate for the full 8 week study period.

Choose the BEST answer.

◯ True (1)

◯ False, my participation is voluntary and I am not required to complete any part of this study and can stop at any time without penalty. (2)

Appendix 1. Nod Consent Form

3.What are some of the possible risks associated with participating in this study?

◯ A. There is a small chance that my participation can cause a loss of privacy. (1)

◯ B. Some of the survey questions or questions within the app may make me feel uncomfortable. (2)

◯ C. There are no risks associated with participating in this study. (3)

◯ D. Both A and B. (4)
