# Supplementary figures and images for: Smartphone App to Address Loneliness Among College Students: Pilot Randomized Controlled Trial
Source: JMIR Ment Health. 2020 Oct 20;7(10):e21496. doi: 10.2196/21496 (PMC7609198; doi:10.2196/21496)

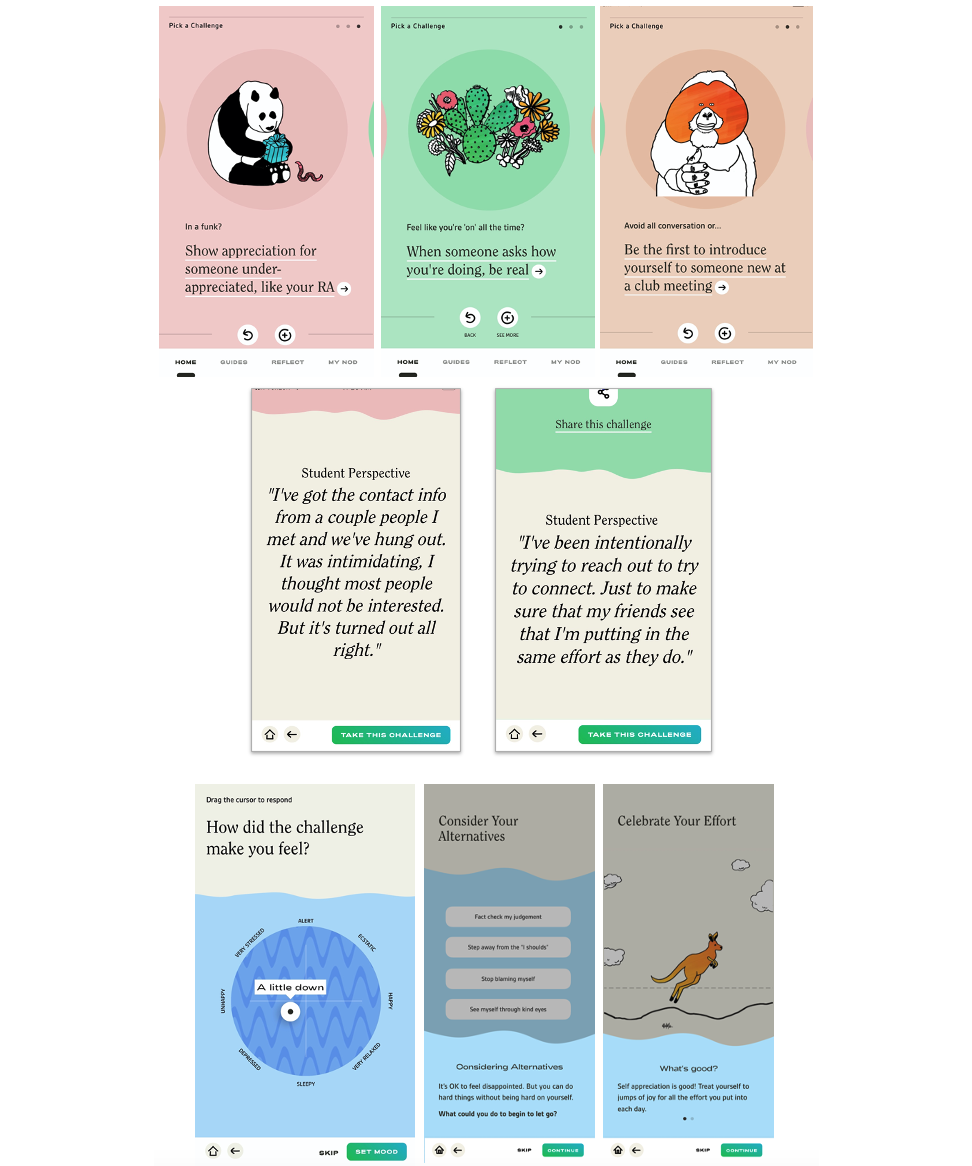

Supplement: Multimedia Appendix 2 [file mental_v7i10e21496_app2.png]
