# Supplementary material for: Smartphone App to Address Loneliness Among College Students: Pilot Randomized Controlled Trial
Source: JMIR Ment Health. 2020 Oct 20;7(10):e21496. doi: 10.2196/21496 (PMC7609198; doi:10.2196/21496)
Supplement: Multimedia Appendix 4 [file mental_v7i10e21496_app4.pdf]

Appendix 4. Nod Risk Assessment

- **Risk Assessment:**

Additionally, if a participant endorses any risk involving self-harm ideation on the PHQ, Qualtrics will be programmed to automatically send a notification via email to Dr. Pfeifer and the project coordinator. If a participant endorses item 9 from PHQ ("Thoughts that you would be better off dead, or of hurting yourself") during any days over the last two-week period, this will trigger the below risk assessment protocol.

The project coordinator would then do the following:

1. Notify Dr. Pfeifer that a notification occurred;
2. Contact the participant via text message and/or email to give campus and other resources for mental health treatment (see "Risk Management Resources" folder);
3. Invite the participant to come to the research lab for a conversation in which the project coordinator can perform further risk assessment and provide additional referrals as appropriate. The project coordinator has been trained in such risk assessment as part of another protocol in DSN lab, Transitions in Adolescent Girls (03232015.027)

Should the participant agree to have a conversation for the purposes of further risk endorsement evaluation, the information gathered will be used to label the risk as medium/low or high. This includes discussion of suicidal ideation, suicidal intent, self-harm intent, plan, lethality, and means. If risk is high the project coordinator will report immediately to Dr. Pfeifer to discuss the appropriate course of action, who will decide based on collected information whether there is evidence that a participant is at risk of serious harm to themselves or someone else. If any cases require further clinical insight, Dr. Pfeifer and the project coordinator will consult with Dr. Nick Allen, a licensed clinical psychologist, to determine the best course of action. It is stated in the plain language statements that confidentiality may be broken in cases where participants may seriously hurt themselves or others, or is mandated by law. For detailed procedure on our decision to break confidentiality in cases of harm, please refer to the risk assessment decision table below.

**Risk Assessment Decision Table**

| **Suicidal Ideation** | Vague | Yes | Yes | No | No | Yes | Yes | Yes |
|---|---|---|---|---|---|---|---|---|
| **Suicidal Intent** | Vague | Vague | Yes | No | No | Yes | Yes | Yes |
| **Self-Harm Intent** | No | No | No | Yes | Yes | No | No | No |
| **Plan** | No | No | No | Yes, but Self Harm only | Yes, but Self Harm only | Yes | Yes | Yes |
| **Lethality** | n/a | n/a | n/a | Low | High | Low | High | High |

Appendix 4. Nod Risk Assessment

| **Means** | n/a | n/a | n/a | Yes | Yes | Yes | Yes | No |
|---|---|---|---|---|---|---|---|---|
| ***Action*** | ***Provide Information*** | | | | ***Break Confidentiality*** | | | |

Qualtrics will also be programmed to send a notification if a participant endorses a score of 1-3 on SACQ item 31 ("I've given a lot of thought lately to whether I should ask for help from Counseling and Psychological Services or from a psychotherapist outside of college"). If a participant endorses 1-3 (out of a 1-9 Likert scale in which 1 = *Applies very close to me* and 9 = *Doesn't apply to me at all*) the project coordinator notify Dr. Pfeifer of the endorsement and then text/email the participant to offer counseling resources with the option to contact the lab for more information, should the participant desire to do so.
