# Supplementary material for: Smartphone App to Address Loneliness Among College Students: Pilot Randomized Controlled Trial
Source: JMIR Ment Health. 2020 Oct 20;7(10):e21496. doi: 10.2196/21496 (PMC7609198; doi:10.2196/21496)

Appendix 5. Comparisons between outcomes and engagement in the control group at week 8 to the experimental group at week 4

Data Analytic Strategy

To validate results, we compared loneliness, mental health, and college adjustment outcomes in the control group at week 8 to those of the experimental group at week 4. Specifically, we analyzed whether there were any main effects of condition, or any condition x baseline vulnerability interactions to predict outcomes after each group had been offered Nod for 4 weeks. These analyses allowed us to explore whether the benefits of Nod were similar irrespective of whether Nod was delivered at the start of participants' first year of college, or four weeks into their first year.

Analyses mirrored those described in the 'data analytic strategy' section of the manuscript, with the exception that we controlled for week 4 scores when predicting week 8 scores in the control group, but controlled for week 0 scores when predicting week 4 scores in the experimental group. This was done to ensure that baseline scores for each group were measured four weeks prior to evaluating outcomes.

Results

Descriptive statistics by condition across the three time points are reported in Table 1 below.

Loneliness

Analyses in Step 1 showed no evidence for an overall effect of condition on loneliness at the end of four weeks of Nod use, $F_{1,210}$=2.47, P=.12. This finding indicates that, on average, students randomly assigned to use Nod four weeks into their first year of college had equivalent outcomes to those randomly assigned to use Nod at the beginning of their first year.

However, participants' depressive symptoms immediately before being assigned to use Nod interacted significantly with condition to predict loneliness at the end of four weeks ($F_{1, 208}$=6.93, P=.009). Examination of simple slopes suggest that Nod was more effective at buffering students with elevated depression against heightened future loneliness when Nod was delivered at the start of the school year than when delivered four weeks into the school year (Table 2; Figure 1).

Mental Health Indicators

Analyses in Step 1 showed no evidence for an overall effect of condition on depression symptoms, anxiety symptoms, or sleep quality at the end of four weeks of Nod use, all Fs <2.0, Ps >.15. However, adjusting for baseline social anxiety symptoms, the experimental group reported significantly lower social anxiety symptoms after four weeks of Nod use than the control, $F_{1,209}$=5.84, P=.02.

In Step 2, loneliness did not significantly moderate the effect of condition on any of the mental health outcomes (all Fs <2.60, Ps >.11).

College Adjustment Indicators

Analyses in Step 1 showed no evidence for an overall effect of condition on perceived social support, campus belonging, or social adjustment after four weeks of Nod use, all Fs <1.3, Ps >.25. Nor were there condition differences in intention to return to college in the subsequent school year (OR=.86, 95% CI [.38-1.95], z=-0.36, P=.72).

However, participants' self-reported loneliness before being assigned Nod significantly interacted with condition to predict social support ($F_{1, 208}$=4.50, P=.035), campus belonging ($F_{1, 207}$=4.49, P=.035), and social adjustment ($F_{1, 208}$=5.85, P=.016) at the end of four weeks of Nod use. Examination of simple slopes suggested that, in all cases, the effects of Nod on college adjustment indicators were more pronounced for lonelier students when Nod was delivered at the start of the school year (Table 2; Figure 1). Likewise, the significant main effect of condition on intention to return was moderated by a condition x baseline loneliness interaction (OR=1.33, 95% CI [1.11-1.59], z=3.07, P=.002). Probing of this interaction revealed that within the control group, the odds of "definitely" intending to return to campus decreased as baseline loneliness increased, even after receipt of Nod. In contrast, in the experimental group the odds of intending to return significantly *increased* as baseline loneliness increased (Table 2).

User Experience

There were no significant differences between the experimental and control groups in the percentage of participants that agreed that Nod was easy to understand ($X^2_{1,210}$=0.22, P=.64), gave them sound advice ($X^2_{1,210}$ =1.07, P=.30), or something new to think about ($X^2_{1,210}$=0.17, P=.68). Nor were there differences in desire to continue to use Nod ($X^2_{1,210}$=2.21, P=.14), or agreement that they had used what they learned in daily life ($X^2_{1,210}$=.32, P=.57) (Figure 2).

Table 1. Means and standard deviations for loneliness, mental health, and college adjustment outcomes at weeks 0, 4,8 among first-year college students in the experimental versus control groups.

**Outcome**	**Week 0**, mean (SD)		**Week 4**, mean (SD)		**Week 8**, mean (SD)	
	Experimental	Control	Experimental	Control	Experimental	Control
Loneliness (UCLA-8)[a]	18.87 (4.32)	18.91 (4.40)	16.71 (4.73)	16.87 (5.32)	16.12 (4.90)	16.43 (5.35)
Depression (PHQ-9)[b]	5.31 (4.18)	6.65 (5.52)	5.71 (4.14)	7.12 (5.90)	5.77 (4.25)	6.89 (5.85)
Anxiety symptoms (GAD-7)[c]	5.90 (4.31)	6.85 (5.10)	5.22 (4.24)	6.50 (5.39)	5.46 (4.34)	6.37 (5.48)
Social anxiety symptoms (Mini-SPIN)[d]	5.21 (2.89)	5.25 (3.23)	4.19 (3.20)	4.54 (3.45)	4.12 (3.03)	4.53 (3.70)
PSQI Sleep quality[e]	1.20 (0.62)	1.33 (0.78)	1.21 (0.64)	1.38 (0.77)	1.27 (0.78)	1.41 (0.87)
Perceived social support (CIT subscale)[f]	not measured	not measured	4.20 (0.67)	4.08 (0.77)	4.15 (0.67)	4.09 (0.73)
Campus belonging (from SERU)[g]	5.00 (0.89)	4.96 (0.89)	4.94 (1.00)	4.86 (0.99)	5.07 (0.87)	4.84 (1.05)
Social adjustment to college (SACQ subscale)[h]	not measured	not measured	6.07 (1.26)	5.92 (1.50)	6.10 (1.34)	5.85 (1.47)

Intention to return (from NSSE)[i]	0.68 (0.47)	0.71 (0.46)	0.69 (0.46)	0.61 (0.49)	0.63 (0.49)	0.62 (0.49)

[a]UCLA-8=UCLA Loneliness Scale, 8-item
[b]PHQ-9=Patient Health Questionnaire, 9-item
[c]GAD-7=Generalized Anxiety Disorder, 7-item scale
[d]Mini-SPIN=Mini Social Phobia Inventory
[e]PSQI Sleep Quality=Sleep Quality item from the Pittsburgh Sleep Quality Index (higher scores reflect lower quality sleep)
[f]CIT=Comprehensive Inventory of Thriving
[g]SERU=Student Experiences in the Research University Questionnaire
[h]SACQ=Student Adaptation to College Questionnaire
[i]NSSE=National Survey for Student Engagement

Table 2. Simple slopes of baseline vulnerability on loneliness and college adjustment outcomes after 4 weeks of Nod use in the experimental versus control groups

**Outcome**	**Group**	***r* of simple slope**	***t* value**	***P* value**
Loneliness (UCLA-8)[a]	Experimental	-0.11	-1.04	.30
	Control	0.20	2.62	.01
Social support (CIT subscale)[b]	Experimental	-0.02	-1.49	.14
	Control	-0.06	-5.55	<.001
Campus belonging (from SERU)[c]	Experimental	0.03	1.50	.13
	Control	-0.02	-1.27	.21
Social Adjustment to College (SACQ subscale)[d]	Experimental	-0.11	-4.08	<.001
	Control	-0.19	-9.53	<.001
Intention to return to college (from NSSE)[e]	Experimental	*OR*[f]=1.19 CI [1.04-1.37]	2.45	.01
	Control	*OR*=0.90, CI [0.80-1.00]	-1.88	.06

[a]UCLA-8 =UCLA Loneliness Scale, 8-item
[b]CIT=Comprehensive Inventory of Thriving;
[c]SERU=Student Experiences in the Research University Questionnaire
[d]SACQ=Student Adaptation to College Questionnaire
[e]NSSE=National Survey for Student Engagement
[f]OR=odds ratio (1=will definitely return; 0=all other responses)

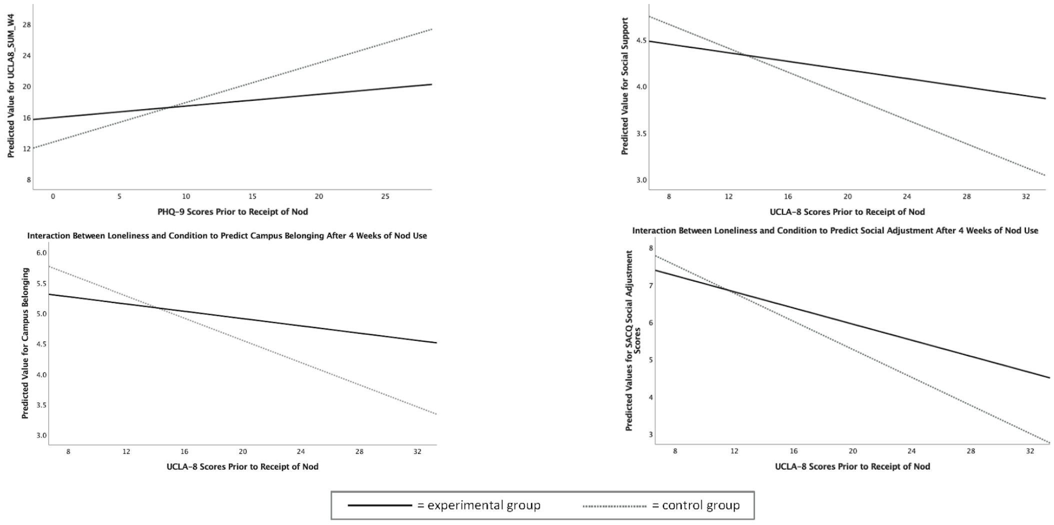

UCLA-8 =UCLA Loneliness Scale, 8-item; PHQ-9=Patient Health Questionnaire 9-item; SACQ = Student Adaptation to College Questionnaire
All figures reflect complete case analyses
Note: higher sleep quality scores = lower sleep quality

Figure 1. Simple slopes of baseline vulnerability on select mental health and college adjustment outcomes in the experimental vs. control groups following 4 weeks of Nod use

Figure 2. Proportion of respondents across the experimental (N=97) and control groups (N=113) who responded 'somewhat agree', 'agree' or 'strongly agree' to the respective items.

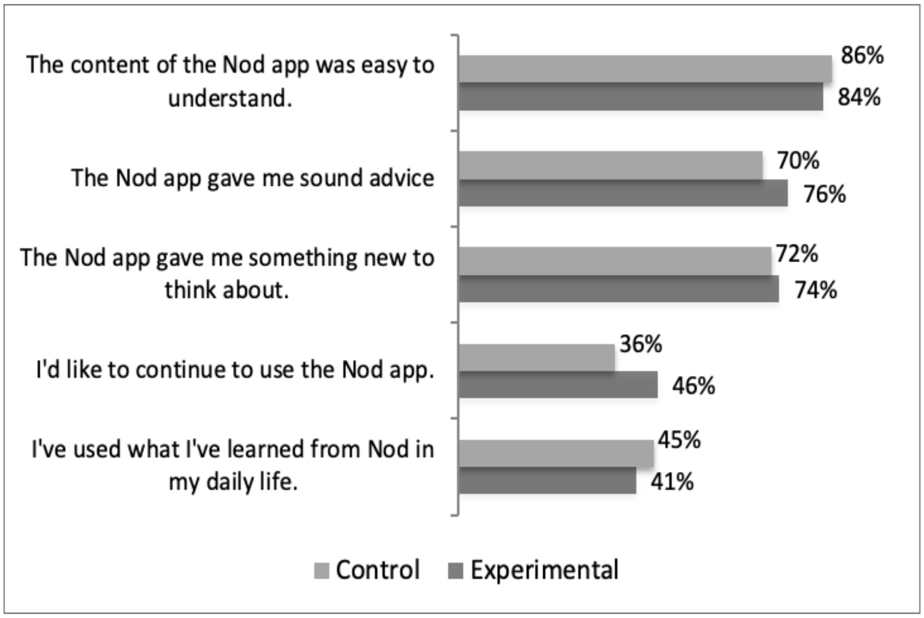

Supplement: Multimedia Appendix 5 [file mental_v7i10e21496_app5.pdf]
