# Supplementary material for: Smartphone App to Address Loneliness Among College Students: Pilot Randomized Controlled Trial
Source: JMIR Ment Health. 2020 Oct 20;7(10):e21496. doi: 10.2196/21496 (PMC7609198; doi:10.2196/21496)
Supplement: Multimedia Appendix 6 [file mental_v7i10e21496_app6.pdf]

CONSORT-EHEALTH (V 1.6.1) - Submission/Publication Form

The CONSORT-EHEALTH checklist is intended for authors of randomized trials evaluating web-based and Internet-based applications/interventions, including mobile interventions, electronic games (incl multiplayer games), social media, certain telehealth applications, and other interactive and/or networked electronic applications. Some of the items (e.g. all subitems under item 5 - description of the intervention) may also be applicable for other study designs.

The goal of the CONSORT EHEALTH checklist and guideline is to be
a) a guide for reporting for authors of RCTs,
b) to form a basis for appraisal of an ehealth trial (in terms of validity)

CONSORT-EHEALTH items/subitems are MANDATORY reporting items for studies published in the Journal of Medical Internet Research and other journals / scientific societies endorsing the checklist.

Items numbered 1., 2., 3., 4a., 4b etc are original CONSORT or CONSORT-NPT (non-pharmacologic treatment) items.
Items with Roman numerals (i., ii, iii, iv etc.) are CONSORT-EHEALTH extensions/clarifications.

As the CONSORT-EHEALTH checklist is still considered in a formative stage, we would ask that you also RATE ON A SCALE OF 1-5 how important/useful you feel each item is FOR THE PURPOSE OF THE CHECKLIST and reporting guideline (optional).

Mandatory reporting items are marked with a red *.
In the textboxes, either copy & paste the relevant sections from your manuscript into this form - please include any quotes from your manuscript in QUOTATION MARKS,
or answer directly by providing additional information not in the manuscript, or elaborating on why the item was not relevant for this study.

YOUR ANSWERS WILL BE PUBLISHED AS A SUPPLEMENTARY FILE TO YOUR PUBLICATION IN JMIR AND ARE CONSIDERED PART OF YOUR PUBLICATION (IF ACCEPTED).
Please fill in these questions diligently. Information will not be copyedited, so please use proper spelling and grammar, use correct capitalization, and avoid abbreviations.

DO NOT FORGET TO SAVE AS PDF _AND_ CLICK THE SUBMIT BUTTON SO YOUR ANSWERS ARE IN OUR DATABASE !!!

Citation Suggestion (if you append the pdf as Appendix we suggest to cite this paper in the caption):
Eysenbach G, CONSORT-EHEALTH Group
CONSORT-EHEALTH: Improving and Standardizing Evaluation Reports of Web-based and

Mobile Health Interventions
J Med Internet Res 2011;13(4):e126
URL: http://www.jmir.org/2011/4/e126/
doi: 10.2196/jmir.1923
PMID: 22209829

* Required

Your name *

First Last

Danielle Ramo

Primary Affiliation (short), City, Country *

University of Toronto, Toronto, Canada

Hopelab

Your e-mail address *

abc@gmail.com

dramo@hopelab.org

Title of your manuscript *

Provide the (draft) title of your manuscript.

Using a Smartphone App to Address Loneliness Among College Students: Pilot Randomized Controlled Trial

Name of your App/Software/Intervention *

If there is a short and a long/alternate name, write the short name first and add the long name in brackets.

Nod

Evaluated Version (if any)

e.g. "V1", "Release 2017-03-01", "Version 2.0.27913"

1.0

Language(s) *

What language is the intervention/app in? If multiple languages are available, separate by comma (e.g. "English, French")

English

URL of your Intervention Website or App

e.g. a direct link to the mobile app on app in appstore (itunes, Google Play), or URL of the website. If the intervention is a DVD or hardware, you can also link to an Amazon page.

http://www.heynod.com

URL of an image/screenshot (optional)

Your answer

Accessibility *

Can an enduser access the intervention presently?

- ○ access is free and open
- ◉ access only for special usergroups, not open
- ○ access is open to everyone, but requires payment/subscription/in-app purchases
- ○ app/intervention no longer accessible
- ○ Other:

Primary Medical Indication/Disease/Condition *

e.g. "Stress", "Diabetes", or define the target group in brackets after the condition, e.g. "Autism (Parents of children with)", "Alzheimers (Informal Caregivers of)"

Loneliness (College Students)

Primary Outcomes measured in trial *

comma-separated list of primary outcomes reported in the trial

Loneliness

Secondary/other outcomes

Are there any other outcomes the intervention is expected to affect?

Depressive symptoms, anxiety symptoms, social anxiety symptoms, sleep quality, perceived social support, campus belonging, social adjustment to college, and intention to remain enrolled

Recommended "Dose" *

What do the instructions for users say on how often the app should be used?

- ( ) Approximately Daily
- ( ) Approximately Weekly
- ( ) Approximately Monthly
- ( ) Approximately Yearly
- (•) "as needed"
- ( ) Other:

Approx. Percentage of Users (starters) still using the app as recommended after 3 months *

- (•) unknown / not evaluated
- ( ) 0-10%
- ( ) 11-20%
- ( ) 21-30%
- ( ) 31-40%
- ( ) 41-50%
- ( ) 51-60%
- ( ) 61-70%
- ( ) 71%-80%
- ( ) 81-90%
- ( ) 91-100%
- ( ) Other:

Overall, was the app/intervention effective? *

- ( ) yes: all primary outcomes were significantly better in intervention group vs control
- ( ) partly: SOME primary outcomes were significantly better in intervention group vs control
- ( ) no statistically significant difference between control and intervention
- ( ) potentially harmful: control was significantly better than intervention in one or more outcomes
- ( ) inconclusive: more research is needed
- (•) Other: Effective for students high in baseline vulnerability

Article Preparation Status/Stage *

At which stage in your article preparation are you currently (at the time you fill in this form)

- ( ) not submitted yet - in early draft status
- ( ) not submitted yet - in late draft status, just before submission
- (•) submitted to a journal but not reviewed yet
- ( ) submitted to a journal and after receiving initial reviewer comments
- ( ) submitted to a journal and accepted, but not published yet
- ( ) published
- ( ) Other:

Journal *

If you already know where you will submit this paper (or if it is already submitted), please provide the journal name (if it is not JMIR, provide the journal name under "other")

- ○ not submitted yet / unclear where I will submit this
- ◉ Journal of Medical Internet Research (JMIR)
- ○ JMIR mHealth and UHealth
- ○ JMIR Serious Games
- ○ JMIR Mental Health
- ○ JMIR Public Health
- ○ JMIR Formative Research
- ○ Other JMIR sister journal
- ○ Other:

Is this a full powered effectiveness trial or a pilot/feasibility trial? *

- ◉ Pilot/feasibility
- ○ Fully powered

Manuscript tracking number *

If this is a JMIR submission, please provide the manuscript tracking number under "other" (The ms tracking number can be found in the submission acknowledgement email, or when you login as author in JMIR. If the paper is already published in JMIR, then the ms tracking number is the four-digit number at the end of the DOI, to be found at the bottom of each published article in JMIR)

- ○ no ms number (yet) / not (yet) submitted to / published in JMIR
- ◉ Other: http://dx.doi.org/10.2196/21496

TITLE AND ABSTRACT

1a) TITLE: Identification as a randomized trial in the title

1a) Does your paper address CONSORT item 1a? *

I.e does the title contain the phrase "Randomized Controlled Trial"? (if not, explain the reason under "other")

- (●) yes
- ( ) Other:

1a-i) Identify the mode of delivery in the title

Identify the mode of delivery. Preferably use “web-based” and/or “mobile” and/or “electronic game” in the title. Avoid ambiguous terms like “online”, “virtual”, “interactive”. Use “Internet-based” only if Intervention includes non-web-based Internet components (e.g. email), use “computer-based” or “electronic” only if offline products are used. Use “virtual” only in the context of “virtual reality” (3-D worlds). Use “online” only in the context of “online support groups”. Complement or substitute product names with broader terms for the class of products (such as “mobile” or “smart phone” instead of “iphone”), especially if the application runs on different platforms.

	1	2	3	4	5	
subitem not at all important	○	○	○	○	○	essential

Does your paper address subitem 1a-i? *

Copy and paste relevant sections from manuscript title (include quotes in quotation marks "like this" to indicate direct quotes from your manuscript), or elaborate on this item by providing additional information not in the ms, or briefly explain why the item is not applicable/relevant for your study

"Using a Smartphone App to Address Loneliness Among College Students"

1a-ii) Non-web-based components or important co-interventions in title

Mention non-web-based components or important co-interventions in title, if any (e.g., “with telephone support”).

	1	2	3	4	5	
subitem not at all important	○	○	○	○	○	essential

Does your paper address subitem 1a-ii?

Copy and paste relevant sections from manuscript title (include quotes in quotation marks "like this" to indicate direct quotes from your manuscript), or elaborate on this item by providing additional information not in the ms, or briefly explain why the item is not applicable/relevant for your study

"Using a Smartphone App to Address Loneliness Among College Students"

1a-iii) Primary condition or target group in the title

Mention primary condition or target group in the title, if any (e.g., “for children with Type I Diabetes”) Example: A Web-based and Mobile Intervention with Telephone Support for Children with Type I Diabetes: Randomized Controlled Trial

	1	2	3	4	5	
subitem not at all important	○	○	○	○	○	essential

Does your paper address subitem 1a-iii? *

Copy and paste relevant sections from manuscript title (include quotes in quotation marks "like this" to indicate direct quotes from your manuscript), or elaborate on this item by providing additional information not in the ms, or briefly explain why the item is not applicable/relevant for your study

"Using a Smartphone App to Address Loneliness Among College Students"

1b) ABSTRACT: Structured summary of trial design, methods, results, and conclusions

NPT extension: Description of experimental treatment, comparator, care providers, centers, and blinding status.

1b-i) Key features/functionalities/components of the intervention and comparator in the METHODS section of the ABSTRACT

Mention key features/functionalities/components of the intervention and comparator in the abstract. If possible, also mention theories and principles used for designing the site. Keep in mind the needs of systematic reviewers and indexers by including important synonyms. (Note: Only report in the abstract what the main paper is reporting. If this information is missing from the main body of text, consider adding it)

	1	2	3	4	5	
subitem not at all important	○	○	○	○	○	essential

Does your paper address subitem 1b-i? *

Copy and paste relevant sections from the manuscript abstract (include quotes in quotation marks "like this" to indicate direct quotes from your manuscript), or elaborate on this item by providing additional information not in the ms, or briefly explain why the item is not applicable/relevant for your study

"The app delivered skills via fully automated (1) “social challenges”--suggested activities designed to build social connections; (2) reflections--brief cognitive reframing exercises and (3) student testimonials that encouraged a growth mindset towards social connection building."

1b-ii) Level of human involvement in the METHODS section of the ABSTRACT

Clarify the level of human involvement in the abstract, e.g., use phrases like “fully automated” vs. “therapist/nurse/care provider/physician-assisted” (mention number and expertise of providers involved, if any). (Note: Only report in the abstract what the main paper is reporting. If this information is missing from the main body of text, consider adding it)

	1	2	3	4	5	
subitem not at all important	○	○	○	○	○	essential

Does your paper address subitem 1b-ii?

Copy and paste relevant sections from the manuscript abstract (include quotes in quotation marks "like this" to indicate direct quotes from your manuscript), or elaborate on this item by providing additional information not in the ms, or briefly explain why the item is not applicable/relevant for your study

"fully automated"

1b-iii) Open vs. closed, web-based (self-assessment) vs. face-to-face assessments in the METHODS section of the ABSTRACT

Mention how participants were recruited (online vs. offline), e.g., from an open access website or from a clinic or a closed online user group (closed usergroup trial), and clarify if this was a purely web-based trial, or there were face-to-face components (as part of the intervention or for assessment). Clearly say if outcomes were self-assessed through questionnaires (as common in web-based trials). Note: In traditional offline trials, an open trial (open-label trial) is a type of clinical trial in which both the researchers and participants know which treatment is being administered. To avoid confusion, use “blinded” or “unblinded” to indicated the level of blinding instead of “open”, as “open” in web-based trials usually refers to “open access” (i.e. participants can self-enrol). (Note: Only report in the abstract what the main paper is reporting. If this information is missing from the main body of text, consider adding it)

	1	2	3	4	5	
subitem not at all important	○	○	○	○	○	essential

Does your paper address subitem 1b-iii?

Copy and paste relevant sections from the manuscript abstract (include quotes in quotation marks "like this" to indicate direct quotes from your manuscript), or elaborate on this item by providing additional information not in the ms, or briefly explain why the item is not applicable/relevant for your study

" First-year college students ... were recruited online via first-year orientation".. "analyses compared the conditions on self-assessed loneliness, depressive symptoms, and other mental health and college adjustment outcomes at week 4"

1b-iv) RESULTS section in abstract must contain use data

Report number of participants enrolled/assessed in each group, the use/uptake of the intervention (e.g., attrition/adherence metrics, use over time, number of logins etc.), in addition to primary/secondary outcomes. (Note: Only report in the abstract what the main paper is reporting. If this information is missing from the main body of text, consider adding it)

	1	2	3	4	5	
subitem not at all important	○	○	○	○	○	essential

Does your paper address subitem 1b-iv?

Copy and paste relevant sections from the manuscript abstract (include quotes in quotation marks "like this" to indicate direct quotes from your manuscript), or elaborate on this item by providing additional information not in the ms, or briefly explain why the item is not applicable/relevant for your study

"participants viewed an average of 36.7 pages of app content"

1b-v) CONCLUSIONS/DISCUSSION in abstract for negative trials

Conclusions/Discussions in abstract for negative trials: Discuss the primary outcome - if the trial is negative (primary outcome not changed), and the intervention was not used, discuss whether negative results are attributable to lack of uptake and discuss reasons. (Note: Only report in the abstract what the main paper is reporting. If this information is missing from the main body of text, consider adding it)

	1	2	3	4	5	
subitem not at all important	○	○	○	○	○	essential

Does your paper address subitem 1b-v?

Copy and paste relevant sections from the manuscript abstract (include quotes in quotation marks "like this" to indicate direct quotes from your manuscript), or elaborate on this item by providing additional information not in the ms, or briefly explain why the item is not applicable/relevant for your study

N/A

INTRODUCTION

2a) In INTRODUCTION: Scientific background and explanation of rationale

2a-i) Problem and the type of system/solution

Describe the problem and the type of system/solution that is object of the study: intended as stand-alone intervention vs. incorporated in broader health care program? Intended for a particular patient population? Goals of the intervention, e.g., being more cost-effective to other interventions, replace or complement other solutions? (Note: Details about the intervention are provided in "Methods" under 5)

	1	2	3	4	5	
subitem not at all important	○	○	○	○	○	essential

Does your paper address subitem 2a-i? *

Copy and paste relevant sections from the manuscript (include quotes in quotation marks "like this" to indicate direct quotes from your manuscript), or elaborate on this item by providing additional information not in the ms, or briefly explain why the item is not applicable/relevant for your study

" Loneliness is especially prevalent among younger generations...[and is] concurrently and prospectively associated with a variety of negative mental health outcomes, including depression, anxiety, social anxiety, and suicidality...Incoming college first-years face a major social transition and may be particularly vulnerable to elevated feelings of loneliness... Although group-based interventions can effectively reduce loneliness, such interventions may have relatively limited reach in university contexts, where mental health centers are often stretched beyond capacity. There is thus a need to develop and test interventions to address loneliness in teens and young adults at scale. Mobile apps offer the ability to deliver mental health resources and interventions in a standardized, scalable and cost-effective manner... Prior research has validated the feasibility and acceptability of smartphone app-based loneliness interventions for young people. More research is needed to evaluate the efficacy of digital interventions targeting loneliness in undergraduate populations."

2a-ii) Scientific background, rationale: What is known about the (type of) system

Scientific background, rationale: What is known about the (type of) system that is the object of the study (be sure to discuss the use of similar systems for other conditions/diagnoses, if appropiate), motivation for the study, i.e. what are the reasons for and what is the context for this specific study, from which stakeholder viewpoint is the study performed, potential impact of findings [2]. Briefly justify the choice of the comparator.

	1	2	3	4	5	
subitem not at all important	○	○	○	○	○	essential

Does your paper address subitem 2a-ii? *

Copy and paste relevant sections from the manuscript (include quotes in quotation marks "like this" to indicate direct quotes from your manuscript), or elaborate on this item by providing additional information not in the ms, or briefly explain why the item is not applicable/relevant for your study

"Loneliness is associated with cognitive biases, including vigilance to social threat and perceptions that others are judging and rejecting.[25,26] Meta-analytic research indicates that the most effective loneliness interventions are those grounded in cognitive behavioral therapy, which target maladaptive cognitions and behaviors.[27] However, most of these interventions have been aimed at older adults, and those aimed at college students tend to be high-touch and resource-intensive. For example, McWhorter & Horan[28] developed an intervention focused on modifying attributional styles with modeling, role playing, and assignments for developing better communication skills. The intervention, consisting of six two-hour structured group experiences led by trained facilitators, significantly decreased participants' loneliness. Although group-based interventions can effectively reduce loneliness, such interventions may have relatively limited reach in university contexts, where mental health centers are often stretched beyond capacity.[29–31] There is thus a need to develop and test interventions to address loneliness in teens and young adults at scale. Mobile apps offer the ability to deliver mental health resources and interventions in a standardized, scalable and cost-effective manner.[32–34] Smartphone ownership is nearly ubiquitous among young adults today,[35] and surveys suggest that nearly one in four smartphone owners aged 18-29 use apps to track or manage health.[36] Further, college counseling centers are increasingly interested in using mHealth applications to disseminate information and interventions to students.[37] Apps provide support on-demand, lowering barriers to much-needed support, such as limited availability of in-person counseling and stigma that can hold back students from seeking help.[37] Prior research has validated the feasibility and acceptability of smartphone app-based loneliness interventions for young people.[38,39] More research is needed to evaluate the efficacy of digital interventions targeting loneliness in undergraduate populations."

2b) In INTRODUCTION: Specific objectives or hypotheses

Does your paper address CONSORT subitem 2b? *

Copy and paste relevant sections from the manuscript (include quotes in quotation marks "like this" to indicate direct quotes from your manuscript), or elaborate on this item by providing additional information not in the ms, or briefly explain why the item is not applicable/relevant for your study

"We present results of a pilot randomized controlled trial of the Nod digital intervention for loneliness among first-year college students (n=221) delivered via smartphone app. Outcomes were compared across two randomly assigned conditions: an experimental group (“Nod”) who received 4 weeks of Nod exposure and a waitlist control group (“control”) who were given access to Nod after 4 weeks. Our primary hypothesis was that students in the Nod group would report lower loneliness by the end of treatment (Week 4) as compared to students in the control group. Secondary hypotheses were that students assigned to use Nod would report better outcomes on key mental health indicators associated with loneliness: depressive symptoms, anxiety symptoms, social anxiety symptoms, and sleep quality. Exploratory analyses examined effects related to friendship and belonging at the university, namely perceived social support, campus belonging, social adjustment to college, and intention to remain enrolled. Finally, we tested the hypothesis that the treatment benefits would be particularly pronounced for students with heightened psychological vulnerability at baseline, given prior research indicating that targeted interventions have greater effect sizes than universal interventions.[40] Since this was a pilot trial, we also examined app engagement and user experience. "

METHODS

3a) Description of trial design (such as parallel, factorial) including allocation ratio

Does your paper address CONSORT subitem 3a? *

Copy and paste relevant sections from the manuscript (include quotes in quotation marks "like this" to indicate direct quotes from your manuscript), or elaborate on this item by providing additional information not in the ms, or briefly explain why the item is not applicable/relevant for your study

"This 4-week pilot randomized controlled trial evaluated the initial efficacy, feasibility, and desirability of Nod. At 4 weeks, control participants were given full app access. An 8-week follow-up survey allowed for validation of the main outcome analyses in the control group, and an exploration of whether uptake of Nod was similar when delivered later in the school year... A total of 415 students were invited to participate. Participants were enrolled on a rolling basis until the target enrollment of 220 students was achieved. This target sample size was selected to allow for the detection of condition differences in week 4 outcomes that were medium in size or larger after accounting for potential loss of participants due to attrition or non-compliance.[42] In total, 221 participants completed a baseline assessment and were randomized to study condition... Following the baseline assessment, participants were randomized via Qualtrics 1:1.2 to either 1) immediate access to Nod (experimental group) or 2) access to Nod following a four-week waiting period (control). Randomization was stratified after dividing students into two groups: higher-loneliness (ie, a mean score ≥21 on the UCLA-8 screening survey, translating to a loneliness score ≥1 SD above the mean of all eligible participants) and lower-loneliness (mean score <21 on the UCLA-8). Twenty more students were recruited into the control group than the experimental group due to the concern that some control participants might access Nod prematurely, and thus need to be excluded from analyses. "

3b) Important changes to methods after trial commencement (such as eligibility criteria), with reasons

Does your paper address CONSORT subitem 3b? *

Copy and paste relevant sections from the manuscript (include quotes in quotation marks "like this" to indicate direct quotes from your manuscript), or elaborate on this item by providing additional information not in the ms, or briefly explain why the item is not applicable/relevant for your study

N/A / none

3b-i) Bug fixes, Downtimes, Content Changes

Bug fixes, Downtimes, Content Changes: ehealth systems are often dynamic systems. A description of changes to methods therefore also includes important changes made on the intervention or comparator during the trial (e.g., major bug fixes or changes in the functionality or content) (5-iii) and other “unexpected events” that may have influenced study design such as staff changes, system failures/downtimes, etc. [2].

	1	2	3	4	5	
subitem not at all important	○	○	○	○	○	essential

Does your paper address subitem 3b-i?

Copy and paste relevant sections from the manuscript (include quotes in quotation marks "like this" to indicate direct quotes from your manuscript), or elaborate on this item by providing additional information not in the ms, or briefly explain why the item is not applicable/relevant for your study

N/A / none

4a) Eligibility criteria for participants

Does your paper address CONSORT subitem 4a? *

Copy and paste relevant sections from the manuscript (include quotes in quotation marks "like this" to indicate direct quotes from your manuscript), or elaborate on this item by providing additional information not in the ms, or briefly explain why the item is not applicable/relevant for your study

"Students were eligible for inclusion if they were: (a) entering their first year of undergraduate education, (b) aged 18-25 years, (c) English literate and (d) not residing with parents/guardians. Students also needed to have a smartphone with an operating system capable of supporting Nod, which >98% of students who met all other four eligibility criteria had."

4a-i) Computer / Internet literacy

Computer / Internet literacy is often an implicit “de facto” eligibility criterion - this should be explicitly clarified.

	1	2	3	4	5	
subitem not at all important	○	○	○	○	○	essential

Does your paper address subitem 4a-i?

Copy and paste relevant sections from the manuscript (include quotes in quotation marks "like this" to indicate direct quotes from your manuscript), or elaborate on this item by providing additional information not in the ms, or briefly explain why the item is not applicable/relevant for your study

"Students also needed to have a smartphone with an operating system capable of supporting Nod, which >98% of students who met all other four eligibility criteria had." It is implied that students need to know how to use the smartphone that they own to participate.

4a-ii) Open vs. closed, web-based vs. face-to-face assessments:

Open vs. closed, web-based vs. face-to-face assessments: Mention how participants were recruited (online vs. offline), e.g., from an open access website or from a clinic, and clarify if this was a purely web-based trial, or there were face-to-face components (as part of the intervention or for assessment), i.e., to what degree got the study team to know the participant. In online-only trials, clarify if participants were quasi-anonymous and whether having multiple identities was possible or whether technical or logistical measures (e.g., cookies, email confirmation, phone calls) were used to detect/prevent these.

	1	2	3	4	5	
subitem not at all important	○	○	○	○	○	essential

Does your paper address subitem 4a-ii? *

Copy and paste relevant sections from the manuscript (include quotes in quotation marks "like this" to indicate direct quotes from your manuscript), or elaborate on this item by providing additional information not in the ms, or briefly explain why the item is not applicable/relevant for your study

"Participants were recruited from July-September 2019, in collaboration with the university's first-year orientation program. All incoming students were asked to indicate if they would like to receive information about a study examining the transition to college via a question embedded within a longer orientation survey. Interested participants (N=2226) were sent additional information, and linked to a brief online screening survey containing questions to assess eligibility, as well as an 8-item version of the UCLA loneliness questionnaire (UCLA-8).... Participants accessed Nod through their university single sign-on, thus preventing an individual from making multiple accounts."

4a-iii) Information giving during recruitment

Information given during recruitment. Specify how participants were briefed for recruitment and in the informed consent procedures (e.g., publish the informed consent documentation as appendix, see also item X26), as this information may have an effect on user self-selection, user expectation and may also bias results.

	1	2	3	4	5	
subitem not at all important	O	O	O	O	O	essential

Does your paper address subitem 4a-iii?

Copy and paste relevant sections from the manuscript (include quotes in quotation marks "like this" to indicate direct quotes from your manuscript), or elaborate on this item by providing additional information not in the ms, or briefly explain why the item is not applicable/relevant for your study

"Participants were recruited from July-September 2019, in collaboration with the university's first-year orientation program. All incoming students were asked to indicate if they would like to receive information about a study examining the transition to college via a question embedded within a longer orientation survey. Interested participants (N=2226) were sent additional information, and a link to a brief online screening survey containing questions to assess eligibility, as well as an 8-item version of the UCLA loneliness questionnaire (UCLA-8)."

4b) Settings and locations where the data were collected

Does your paper address CONSORT subitem 4b? *

Copy and paste relevant sections from the manuscript (include quotes in quotation marks "like this" to indicate direct quotes from your manuscript), or elaborate on this item by providing additional information not in the ms, or briefly explain why the item is not applicable/relevant for your study

"Participants were incoming first-year students at a large public university in the Northwestern U.S" / study was conducted online

4b-i) Report if outcomes were (self-)assessed through online questionnaires

Clearly report if outcomes were (self-)assessed through online questionnaires (as common in web-based trials) or otherwise.

	1	2	3	4	5	
subitem not at all important	○	○	○	○	○	essential

Does your paper address subitem 4b-i? *

Copy and paste relevant sections from the manuscript (include quotes in quotation marks "like this" to indicate direct quotes from your manuscript), or elaborate on this item by providing additional information not in the ms, or briefly explain why the item is not applicable/relevant for your study

"Online assessments through Qualtrics Software (Salt Lake City, UT) were administered at baseline and at weeks 2, 4, and 8. Participants received US $20 gift cards for (a) creating a Nod account within one week of receiving the email invitation, (b) completing each of four surveys ($20/survey), and (c) completing all four surveys (a $20 bonus), for a possible total of US $120 for their participation. "

4b-ii) Report how institutional affiliations are displayed

Report how institutional affiliations are displayed to potential participants [on ehealth media], as affiliations with prestigious hospitals or universities may affect volunteer rates, use, and reactions with regards to an intervention.(Not a required item – describe only if this may bias results)

	1	2	3	4	5	
subitem not at all important	○	○	○	○	○	essential

Does your paper address subitem 4b-ii?

Copy and paste relevant sections from the manuscript (include quotes in quotation marks "like this" to indicate direct quotes from your manuscript), or elaborate on this item by providing additional information not in the ms, or briefly explain why the item is not applicable/relevant for your study

N/A

5) The interventions for each group with sufficient details to allow replication, including how and when they were actually administered

5-i) Mention names, credential, affiliations of the developers, sponsors, and owners

Mention names, credential, affiliations of the developers, sponsors, and owners [6] (if authors/evaluators are owners or developer of the software, this needs to be declared in a “Conflict of interest” section or mentioned elsewhere in the manuscript).

	1	2	3	4	5	
subitem not at all important	○	○	○	○	○	essential

Does your paper address subitem 5-i?

Copy and paste relevant sections from the manuscript (include quotes in quotation marks "like this" to indicate direct quotes from your manuscript), or elaborate on this item by providing additional information not in the ms, or briefly explain why the item is not applicable/relevant for your study

"Nod is a mobile app co-developed by Grit Digital Health and Hopelab. While both organizations own rights to the Nod product, Grit Digital Health is solely responsible for the commercial operation and distribution of Nod. As co-owner, Hopelab receives a limited portion of Nod sale net proceeds. The research reported here as well as the development of Nod was supported by the nonprofit Hopelab Foundation. Hopelab develops behavioral interventions to improve the health and well-being of young people. The design, conduct, analysis, and reporting of this study represents a scientific collaboration between Hopelab, Jennifer Pfeifer, and Brittany Davis at the University of Oregon, and Kevin Delucchi at the University of California, San Francisco and Weill Institute for Neurosciences. Emma Bruehlman-Senecal, Cayce Hook, Caroline Fitzgerald, Jana Haritatos, and Danielle Ramo are employed by Hopelab Foundation. The study sponsor was involved in the study design, collection, analysis, and interpretation of data, writing of the article, and decision to submit it for publication."

5-ii) Describe the history/development process

Describe the history/development process of the application and previous formative evaluations (e.g., focus groups, usability testing), as these will have an impact on adoption/use rates and help with interpreting results.

	1	2	3	4	5	
subitem not at all important	○	○	○	○	○	essential

Does your paper address subitem 5-ii?

Copy and paste relevant sections from the manuscript (include quotes in quotation marks "like this" to indicate direct quotes from your manuscript), or elaborate on this item by providing additional information not in the ms, or briefly explain why the item is not applicable/relevant for your study

"Before launching the pilot trial, Hopelab conducted formative work through interviews, focus groups, and surveys of first-year college students. The app content and visual elements were tailored based on student feedback."

5-iii) Revisions and updating

Revisions and updating. Clearly mention the date and/or version number of the application/intervention (and comparator, if applicable) evaluated, or describe whether the intervention underwent major changes during the evaluation process, or whether the development and/or content was “frozen” during the trial. Describe dynamic components such as news feeds or changing content which may have an impact on the replicability of the intervention (for unexpected events see item 3b).

	1	2	3	4	5	
subitem not at all important	○	○	○	○	○	essential

Does your paper address subitem 5-iii?

Copy and paste relevant sections from the manuscript (include quotes in quotation marks "like this" to indicate direct quotes from your manuscript), or elaborate on this item by providing additional information not in the ms, or briefly explain why the item is not applicable/relevant for your study

N/A

5-iv) Quality assurance methods

Provide information on quality assurance methods to ensure accuracy and quality of information provided [1], if applicable.

	1	2	3	4	5	
subitem not at all important	○	○	○	○	○	essential

Does your paper address subitem 5-iv?

Copy and paste relevant sections from the manuscript (include quotes in quotation marks "like this" to indicate direct quotes from your manuscript), or elaborate on this item by providing additional information not in the ms, or briefly explain why the item is not applicable/relevant for your study

"Analyses of all outcome variables utilized an ‘intent-to-treat approach’, which included all available data from participants randomly assigned to the experimental and control groups."

5-v) Ensure replicability by publishing the source code, and/or providing screenshots/screen-capture video, and/or providing flowcharts of the algorithms used

Ensure replicability by publishing the source code, and/or providing screenshots/screen-capture video, and/or providing flowcharts of the algorithms used. Replicability (i.e., other researchers should in principle be able to replicate the study) is a hallmark of scientific reporting.

	1	2	3	4	5	
subitem not at all important	○	○	○	○	○	essential

Does your paper address subitem 5-v?

Copy and paste relevant sections from the manuscript (include quotes in quotation marks "like this" to indicate direct quotes from your manuscript), or elaborate on this item by providing additional information not in the ms, or briefly explain why the item is not applicable/relevant for your study

Your answer

5-vi) Digital preservation

Digital preservation: Provide the URL of the application, but as the intervention is likely to change or disappear over the course of the years; also make sure the intervention is archived (Internet Archive, webcitation.org, and/or publishing the source code or screenshots/videos alongside the article). As pages behind login screens cannot be archived, consider creating demo pages which are accessible without login.

	1	2	3	4	5	
subitem not at all important	○	○	○	○	○	essential

Does your paper address subitem 5-vi?

Copy and paste relevant sections from the manuscript (include quotes in quotation marks "like this" to indicate direct quotes from your manuscript), or elaborate on this item by providing additional information not in the ms, or briefly explain why the item is not applicable/relevant for your study

Your answer

5-vii) Access

Access: Describe how participants accessed the application, in what setting/context, if they had to pay (or were paid) or not, whether they had to be a member of specific group. If known, describe how participants obtained “access to the platform and Internet” [1]. To ensure access for editors/reviewers/readers, consider to provide a “backdoor” login account or demo mode for reviewers/readers to explore the application (also important for archiving purposes, see vi).

	1	2	3	4	5	
subitem not at all important	○	○	○	○	○	essential

Does your paper address subitem 5-vii? *

Copy and paste relevant sections from the manuscript (include quotes in quotation marks "like this" to indicate direct quotes from your manuscript), or elaborate on this item by providing additional information not in the ms, or briefly explain why the item is not applicable/relevant for your study

"Within 72 hours of completing the baseline survey, participants in the experimental group were emailed an invitation to download Nod. Those in the control group were notified via email that they had been randomly selected to receive access to the app after four weeks, and advised to await a download invitation.
Online assessments through Qualtrics Software (Salt Lake City, UT) were administered at baseline and at weeks 2, 4, and 8. Participants received US $20 Amazon gift cards for (a) creating a Nod account within one week of receiving the email invitation, (b) completing each of four surveys ($20/survey), and (c) completing all four surveys (a $20 bonus), for a possible total of US $120 for their participation. Participants were not incentivized for app usage beyond account registration, and were instructed to use Nod as much or as little as they desired."

5-viii) Mode of delivery, features/functionalities/components of the intervention and comparator, and the theoretical framework

Describe mode of delivery, features/functionalities/components of the intervention and comparator, and the theoretical framework [6] used to design them (instructional strategy [1], behaviour change techniques, persuasive features, etc., see e.g., [7, 8] for terminology). This includes an in-depth description of the content (including where it is coming from and who developed it) [1],” whether [and how] it is tailored to individual circumstances and allows users to track their progress and receive feedback” [6]. This also includes a description of communication delivery channels and – if computer-mediated communication is a component – whether communication was synchronous or asynchronous [6]. It also includes information on presentation strategies [1], including page design principles, average amount of text on pages, presence of hyperlinks to other resources, etc. [1].

	1	2	3	4	5	
subitem not at all important	○	○	○	○	○	essential

Does your paper address subitem 5-viii? *

Copy and paste relevant sections from the manuscript (include quotes in quotation marks "like this" to indicate direct quotes from your manuscript), or elaborate on this item by providing additional information not in the ms, or briefly explain why the item is not applicable/relevant for your study

"Nod is a mobile app that incorporates positive psychology, mindfulness-based selfcompassion,
and cognitive behavioral skill building exercises to address loneliness among
first-year college students. Nod delivers skills via three key features: (1) social challenges—suggested campus-based social activities designed to build social connections; (2) reflections—short in-app exercises that help students process social experiences and reduce self-criticism; and (3) student testimonials that encourage a growth mindset towards social connection building. These features were based on exercises and interventions demonstrated to build social connectedness and address negative self and social cognitions in prior empirical research.
Social challenge content focused on six core social skills and behaviors known to build and strengthen social connections: (1) performing acts of kindness,[44,45] (2) expressing gratitude,[46,47] (3) active listening,[48,49] (4) initiating social outreach/invitations,[50,51] (5) being receptive to others' invitations, and (6) engaging in appropriate self-disclosure.[52,53]
Reflections were short in-app exercises designed to scaffold cognitive restructuring of negative social experiences and savoring of positive social experiences. After completing app-based social challenges, participants were directed to use an interactive mood rating tool to indicate how they felt about their social experience. Positive mood ratings directed participants to exercises designed to amplify and prolong positive emotions, such as savoring[54] and gratitude.[46] Negative ratings directed participants to cognitive reframing exercises such as self-compassion meditations [55,56] and reappraisal.[57,58]. Participants could also select "stand-alone" reflection exercises, which provided guided reflection around social experiences encountered in everyday life.
To reinforce a growth mindset towards college friendship [59–61], challenges were accompanied by brief testimonials (ie, short recommendations of specific in-app social challenges written by college students), which were selected to support the belief that forming satisfying social connections takes time and effort.
Before launching the pilot trial, Hopelab conducted formative work through interviews, focus groups, and surveys of first-year college students. The app content and visual elements were tailored based on student feedback. Content and development of Nod 1.0 were frozen during the trial. Examples of challenge, reflection, and student testimonial content are outlined in Multimedia Appendix 1."

5-ix) Describe use parameters

Describe use parameters (e.g., intended “doses” and optimal timing for use). Clarify what instructions or recommendations were given to the user, e.g., regarding timing, frequency, heaviness of use, if any, or was the intervention used ad libitum.

	1	2	3	4	5	
subitem not at all important	○	○	○	○	○	essential

Does your paper address subitem 5-ix?

Copy and paste relevant sections from the manuscript (include quotes in quotation marks "like this" to indicate direct quotes from your manuscript), or elaborate on this item by providing additional information not in the ms, or briefly explain why the item is not applicable/relevant for your study

Your answer

5-x) Clarify the level of human involvement

Clarify the level of human involvement (care providers or health professionals, also technical assistance) in the e-intervention or as co-intervention (detail number and expertise of professionals involved, if any, as well as “type of assistance offered, the timing and frequency of the support, how it is initiated, and the medium by which the assistance is delivered”. It may be necessary to distinguish between the level of human involvement required for the trial, and the level of human involvement required for a routine application outside of a RCT setting (discuss under item 21 – generalizability).

	1	2	3	4	5	
subitem not at all important	○	○	○	○	○	essential

Does your paper address subitem 5-x?

Copy and paste relevant sections from the manuscript (include quotes in quotation marks "like this" to indicate direct quotes from your manuscript), or elaborate on this item by providing additional information not in the ms, or briefly explain why the item is not applicable/relevant for your study

Your answer

5-xi) Report any prompts/reminders used

Report any prompts/reminders used: Clarify if there were prompts (letters, emails, phone calls, SMS) to use the application, what triggered them, frequency etc. It may be necessary to distinguish between the level of prompts/reminders required for the trial, and the level of prompts/reminders for a routine application outside of a RCT setting (discuss under item 21 – generalizability).

	1	2	3	4	5	
subitem not at all important	○	○	○	○	○	essential

Does your paper address subitem 5-xi? *

Copy and paste relevant sections from the manuscript (include quotes in quotation marks "like this" to indicate direct quotes from your manuscript), or elaborate on this item by providing additional information not in the ms, or briefly explain why the item is not applicable/relevant for your study

N/A / none

5-xii) Describe any co-interventions (incl. training/support)

Describe any co-interventions (incl. training/support): Clearly state any interventions that are provided in addition to the targeted eHealth intervention, as ehealth intervention may not be designed as stand-alone intervention. This includes training sessions and support [1]. It may be necessary to distinguish between the level of training required for the trial, and the level of training for a routine application outside of a RCT setting (discuss under item 21 – generalizability.

	1	2	3	4	5	
subitem not at all important	○	○	○	○	○	essential

Does your paper address subitem 5-xii? *

Copy and paste relevant sections from the manuscript (include quotes in quotation marks "like this" to indicate direct quotes from your manuscript), or elaborate on this item by providing additional information not in the ms, or briefly explain why the item is not applicable/relevant for your study

N/A / none

6a) Completely defined pre-specified primary and secondary outcome measures, including how and when they were assessed

Does your paper address CONSORT subitem 6a? *

Copy and paste relevant sections from the manuscript (include quotes in quotation marks "like this" to indicate direct quotes from your manuscript), or elaborate on this item by providing additional information not in the ms, or briefly explain why the item is not applicable/relevant for your study

"Our primary hypothesis was that students in the Nod group would report lower loneliness by the end of treatment (Week 4) as compared to students in the control group. Secondary hypotheses were that students assigned to use Nod would report better outcomes on key mental health indicators associated with loneliness: depressive symptoms, anxiety symptoms, social anxiety symptoms, and sleep quality."

6a-i) Online questionnaires: describe if they were validated for online use and apply CHERRIES items to describe how the questionnaires were designed/deployed

If outcomes were obtained through online questionnaires, describe if they were validated for online use and apply CHERRIES items to describe how the questionnaires were designed/deployed [9].

	1	2	3	4	5	
subitem not at all important	◉	○	○	○	○	essential

Does your paper address subitem 6a-i?

Copy and paste relevant sections from manuscript text

Your answer

6a-ii) Describe whether and how “use” (including intensity of use/dosage) was defined/measured/monitored

Describe whether and how “use” (including intensity of use/dosage) was defined/measured/monitored (logins, logfile analysis, etc.). Use/adoption metrics are important process outcomes that should be reported in any ehealth trial.

	1	2	3	4	5	
subitem not at all important	○	○	○	○	○	essential

Does your paper address subitem 6a-ii?

Copy and paste relevant sections from manuscript text

Your answer

6a-iii) Describe whether, how, and when qualitative feedback from participants was obtained

Describe whether, how, and when qualitative feedback from participants was obtained (e.g., through emails, feedback forms, interviews, focus groups).

	1	2	3	4	5	
subitem not at all important	○	○	○	○	○	essential

Does your paper address subitem 6a-iii?

Copy and paste relevant sections from manuscript text

Your answer

6b) Any changes to trial outcomes after the trial commenced, with reasons

Does your paper address CONSORT subitem 6b? *

Copy and paste relevant sections from the manuscript (include quotes in quotation marks "like this" to indicate direct quotes from your manuscript), or elaborate on this item by providing additional information not in the ms, or briefly explain why the item is not applicable/relevant for your study

N/A no changes

7a) How sample size was determined

NPT: When applicable, details of whether and how the clustering by care provides or centers was addressed

7a-i) Describe whether and how expected attrition was taken into account when calculating the sample size

Describe whether and how expected attrition was taken into account when calculating the sample size.

	1	2	3	4	5	
subitem not at all important	○	○	○	○	○	essential

Does your paper address subitem 7a-i?

Copy and paste relevant sections from manuscript title (include quotes in quotation marks "like this" to indicate direct quotes from your manuscript), or elaborate on this item by providing additional information not in the ms, or briefly explain why the item is not applicable/relevant for your study

Your answer

7b) When applicable, explanation of any interim analyses and stopping guidelines

Does your paper address CONSORT subitem 7b? *

Copy and paste relevant sections from the manuscript (include quotes in quotation marks "like this" to indicate direct quotes from your manuscript), or elaborate on this item by providing additional information not in the ms, or briefly explain why the item is not applicable/relevant for your study

N/A / none

8a) Method used to generate the random allocation sequence

NPT: When applicable, how care providers were allocated to each trial group

Does your paper address CONSORT subitem 8a? *

Copy and paste relevant sections from the manuscript (include quotes in quotation marks "like this" to indicate direct quotes from your manuscript), or elaborate on this item by providing additional information not in the ms, or briefly explain why the item is not applicable/relevant for your study

"participants were randomized via Qualtrics 1:1.2"

8b) Type of randomisation; details of any restriction (such as blocking and block size)

Does your paper address CONSORT subitem 8b? *

Copy and paste relevant sections from the manuscript (include quotes in quotation marks "like this" to indicate direct quotes from your manuscript), or elaborate on this item by providing additional information not in the ms, or briefly explain why the item is not applicable/relevant for your study

"Randomization was stratified after dividing students into two groups: higher-loneliness (ie, a mean score ≥21 on the UCLA-8 screening survey, translating to a loneliness score ≥1 SD above the mean of all eligible participants) and lower-loneliness (mean score <21 on the UCLA-8)."

9) Mechanism used to implement the random allocation sequence (such as sequentially numbered containers), describing any steps taken to conceal the sequence until interventions were assigned

Does your paper address CONSORT subitem 9? *

Copy and paste relevant sections from the manuscript (include quotes in quotation marks "like this" to indicate direct quotes from your manuscript), or elaborate on this item by providing additional information not in the ms, or briefly explain why the item is not applicable/relevant for your study

"participants were randomized via Qualtrics 1:1.2"

10) Who generated the random allocation sequence, who enrolled participants, and who assigned participants to interventions

Does your paper address CONSORT subitem 10? *

Copy and paste relevant sections from the manuscript (include quotes in quotation marks "like this" to indicate direct quotes from your manuscript), or elaborate on this item by providing additional information not in the ms, or briefly explain why the item is not applicable/relevant for your study

"participants were randomized via Qualtrics 1:1.2"

11a) If done, who was blinded after assignment to interventions (for example, participants, care providers, those assessing outcomes) and how

NPT: Whether or not administering co-interventions were blinded to group assignment

11a-i) Specify who was blinded, and who wasn't

Specify who was blinded, and who wasn't. Usually, in web-based trials it is not possible to blind the participants [1, 3] (this should be clearly acknowledged), but it may be possible to blind outcome assessors, those doing data analysis or those administering co-interventions (if any).

	1	2	3	4	5	
subitem not at all important	○	○	○	○	○	essential

Does your paper address subitem 11a-i? *

Copy and paste relevant sections from the manuscript (include quotes in quotation marks "like this" to indicate direct quotes from your manuscript), or elaborate on this item by providing additional information not in the ms, or briefly explain why the item is not applicable/relevant for your study

Participants were not blind (waitlist control). Blinding not an issue for outcome assessment because self-assessments were used. Staff doing data analysis were not blinded

11a-ii) Discuss e.g., whether participants knew which intervention was the "intervention of interest" and which one was the "comparator"

Informed consent procedures (4a-ii) can create biases and certain expectations - discuss e.g., whether participants knew which intervention was the "intervention of interest" and which one was the "comparator".

	1	2	3	4	5	
subitem not at all important	○	○	○	○	○	essential

Does your paper address subitem 11a-ii?

Copy and paste relevant sections from the manuscript (include quotes in quotation marks "like this" to indicate direct quotes from your manuscript), or elaborate on this item by providing additional information not in the ms, or briefly explain why the item is not applicable/relevant for your study

Your answer

11b) If relevant, description of the similarity of interventions

(this item is usually not relevant for ehealth trials as it refers to similarity of a placebo or sham intervention to a active medication/intervention)

Does your paper address CONSORT subitem 11b? *

Copy and paste relevant sections from the manuscript (include quotes in quotation marks "like this" to indicate direct quotes from your manuscript), or elaborate on this item by providing additional information not in the ms, or briefly explain why the item is not applicable/relevant for your study

N/A; waitlist control

12a) Statistical methods used to compare groups for primary and secondary outcomes

NPT: When applicable, details of whether and how the clustering by care providers or centers was addressed

Does your paper address CONSORT subitem 12a? *

Copy and paste relevant sections from the manuscript (include quotes in quotation marks "like this" to indicate direct quotes from your manuscript), or elaborate on this item by providing additional information not in the ms, or briefly explain why the item is not applicable/relevant for your study

"Analyses of all outcome variables utilized an 'intent-to-treat approach', which included all available data from participants randomly assigned to the experimental and control groups. We took a two-step approach to these analyses, reflecting our two main lines of inquiry. In Step 1, we tested the primary and secondary hypotheses that the experimental group would report lower loneliness, and other indicators of better mental health and college adjustment at the end of treatment (week 4) as compared to the control group. In Step 2, we tested the hypothesis that treatment benefits would be more pronounced for participants with heightened psychological vulnerability at baseline.
Step 1 evaluated condition differences in outcomes at the end of treatment (week 4). Because missing data at week 4 was minimal (213/221, 96% of the sample provided full data on all outcome variables) we opted for a straightforward analytic approach that compared the means of the experimental and control groups on each outcome at week 4, adjusting for each outcome's respective baseline value. A separate ANCOVA was conducted for each outcome, and each model was evaluated on the basis of the statistical significance (P<.05) of the condition term (1=experimental; 0=control). Two outcomes, 'social adjustment to college' and 'perceived social support', were not measured at baseline, because participants had not yet had enough social experiences on campus to meaningfully answer survey questions. Thus, models for these two outcomes omit baseline scores as a covariate.
Step 2 added an interaction term between baseline vulnerability and condition, allowing us to evaluate whether the benefits of Nod were more pronounced for more vulnerable students. The model of loneliness at week 4 included four predictors: condition, baseline loneliness, baseline depression, and a condition X baseline depression interaction term to capture baseline vulnerability. In modeling all other outcomes, models included four predictors: condition, baseline loneliness, baseline score on the outcome variable, and a condition X baseline loneliness interaction term. We selected depression as the baseline moderator of week 4 loneliness, and loneliness as the baseline moderator of week 4 depression and all other outcomes, given previous research demonstrating a strong bivariate and reciprocal relationship between loneliness and depression [5,9,74], including in first year college students,[74] and a strong relationship at baseline in the present study (r=.52). To determine whether Nod differentially benefitted vulnerable participants, each model was evaluated on the basis of the statistical significance (P<.05) of the interaction term."

12a-i) Imputation techniques to deal with attrition / missing values

Imputation techniques to deal with attrition / missing values: Not all participants will use the intervention/comparator as intended and attrition is typically high in ehealth trials. Specify how participants who did not use the application or dropped out from the trial were treated in the statistical analysis (a complete case analysis is strongly discouraged, and simple imputation techniques such as LOCF may also be problematic [4]).

	1	2	3	4	5	
subitem not at all important	○	○	○	○	○	essential

Does your paper address subitem 12a-i? *

Copy and paste relevant sections from the manuscript (include quotes in quotation marks "like this" to indicate direct quotes from your manuscript), or elaborate on this item by providing additional information not in the ms, or briefly explain why the item is not applicable/relevant for your study

"Analyses of all outcome variables utilized an ‘intent-to-treat approach’, which included all available data from participants randomly assigned to the experimental and control groups.
"

12b) Methods for additional analyses, such as subgroup analyses and adjusted analyses

Does your paper address CONSORT subitem 12b? *

Copy and paste relevant sections from the manuscript (include quotes in quotation marks "like this" to indicate direct quotes from your manuscript), or elaborate on this item by providing additional information not in the ms, or briefly explain why the item is not applicable/relevant for your study

"Analyses of all outcome variables utilized an ‘intent-to-treat approach’, which included all available data from participants randomly assigned to the experimental and control groups. We took a two-step approach to these analyses, reflecting our two main lines of inquiry. In Step 1, we tested the primary and secondary hypotheses that the experimental group would report lower loneliness, and other indicators of better mental health and college adjustment at the end of treatment (week 4) as compared to the control group. In Step 2, we tested the hypothesis that treatment benefits would be more pronounced for participants with heightened psychological vulnerability at baseline.
Step 1 evaluated condition differences in outcomes at the end of treatment (week 4). Because missing data at week 4 was minimal (213/221, 96% of the sample provided full data on all outcome variables) we opted for a straightforward analytic approach that compared the means of the experimental and control groups on each outcome at week 4, adjusting for each outcome’s respective baseline value. A separate ANCOVA was conducted for each outcome, and each model was evaluated on the basis of the statistical significance (P<.05) of the condition term (1=experimental; 0=control). Two outcomes, ‘social adjustment to college’ and ‘perceived social support’, were not measured at baseline, because participants had not yet had enough social experiences on campus to meaningfully answer survey questions. Thus, models for these two outcomes omit baseline scores as a covariate.
Step 2 added an interaction term between baseline vulnerability and condition, allowing us to evaluate whether the benefits of Nod were more pronounced for more vulnerable students. The model of loneliness at week 4 included four predictors: condition, baseline loneliness, baseline depression, and a condition X baseline depression interaction term to capture baseline vulnerability. In modeling all other outcomes, models included four predictors: condition, baseline loneliness, baseline score on the outcome variable, and a condition X baseline loneliness interaction term. We selected depression as the baseline moderator of week 4 loneliness, and loneliness as the baseline moderator of week 4 depression and all other outcomes, given previous research demonstrating a strong bivariate and reciprocal relationship between loneliness and depression [5,9,74], including in first year college students,[74] and a strong relationship at baseline in the present study (r=.52). To determine whether Nod differentially benefitted vulnerable participants, each model was evaluated on the basis of the statistical significance (P<.05) of the interaction term."

X26) REB/IRB Approval and Ethical Considerations [recommended as subheading under "Methods"] (not a CONSORT item)

X26-i) Comment on ethics committee approval

	1	2	3	4	5	
subitem not at all important	○	○	○	○	○	essential

Does your paper address subitem X26-i?

Copy and paste relevant sections from the manuscript (include quotes in quotation marks "like this" to indicate direct quotes from your manuscript), or elaborate on this item by providing additional information not in the ms, or briefly explain why the item is not applicable/relevant for your study

Your answer

x26-ii) Outline informed consent procedures

Outline informed consent procedures e.g., if consent was obtained offline or online (how? Checkbox, etc.?), and what information was provided (see 4a-ii). See [6] for some items to be included in informed consent documents.

	1	2	3	4	5	
subitem not at all important	○	○	○	○	○	essential

Does your paper address subitem X26-ii?

Copy and paste relevant sections from the manuscript (include quotes in quotation marks "like this" to indicate direct quotes from your manuscript), or elaborate on this item by providing additional information not in the ms, or briefly explain why the item is not applicable/relevant for your study

Your answer

X26-iii) Safety and security procedures

Safety and security procedures, incl. privacy considerations, and any steps taken to reduce the likelihood or detection of harm (e.g., education and training, availability of a hotline)

	1	2	3	4	5	
subitem not at all important	○	○	○	○	○	essential

Does your paper address subitem X26-iii?

Copy and paste relevant sections from the manuscript (include quotes in quotation marks "like this" to indicate direct quotes from your manuscript), or elaborate on this item by providing additional information not in the ms, or briefly explain why the item is not applicable/relevant for your study

Your answer

RESULTS

13a) For each group, the numbers of participants who were randomly assigned, received intended treatment, and were analysed for the primary outcome

NPT: The number of care providers or centers performing the intervention in each group and the number of patients treated by each care provider in each center

Does your paper address CONSORT subitem 13a? *

Copy and paste relevant sections from the manuscript (include quotes in quotation marks "like this" to indicate direct quotes from your manuscript), or elaborate on this item by providing additional information not in the ms, or briefly explain why the item is not applicable/relevant for your study

Figure 1; Table 3

13b) For each group, losses and exclusions after randomisation, together with reasons

Does your paper address CONSORT subitem 13b? (NOTE: Preferably, this is shown in a CONSORT flow diagram) *

Copy and paste relevant sections from the manuscript (include quotes in quotation marks "like this" to indicate direct quotes from your manuscript), or elaborate on this item by providing additional information not in the ms, or briefly explain why the item is not applicable/relevant for your study

Figure 1

13b-i) Attrition diagram

Strongly recommended: An attrition diagram (e.g., proportion of participants still logging in or using the intervention/comparator in each group plotted over time, similar to a survival curve) or other figures or tables demonstrating usage/dose/engagement.

	1	2	3	4	5	
subitem not at all important	○	○	○	○	○	essential

Does your paper address subitem 13b-i?

Copy and paste relevant sections from the manuscript or cite the figure number if applicable (include quotes in quotation marks "like this" to indicate direct quotes from your manuscript), or elaborate on this item by providing additional information not in the ms, or briefly explain why the item is not applicable/relevant for your study

Your answer

14a) Dates defining the periods of recruitment and follow-up

Does your paper address CONSORT subitem 14a? *

Copy and paste relevant sections from the manuscript (include quotes in quotation marks "like this" to indicate direct quotes from your manuscript), or elaborate on this item by providing additional information not in the ms, or briefly explain why the item is not applicable/relevant for your study

Participants were recruited from July-September 2019, in collaboration with the university's first-year orientation program. All incoming students indicated whether they would like to receive information about a study examining the college transition via a question embedded within a longer orientation survey. Interested participants (N=2226) were sent additional information, and linked to a brief online screening survey containing questions to assess eligibility, as well as an 8-item version of the UCLA loneliness questionnaire (UCLA-8). Informed consent was obtained online, with assessment of understanding used in previous online research,[43] immediately prior to completion of the baseline assessment. Following the baseline assessment, participants were randomized via Qualtrics 1:1.2 to either 1) immediate access to Nod (experimental group) or 2) access to Nod following a four-week waiting period (control). Randomization was stratified after dividing students into two groups: higher-loneliness (ie, a mean score ≥21 on the UCLA-8 screening survey, translating to a loneliness score ≥1 SD above the mean of all eligible participants) and lower-loneliness (mean score <21 on the UCLA-8). Twenty more students were recruited into the control group due to the concern that some control participants might need to be excluded from analyses for accessing Nod prematurely. In-app data confirmed that no control participants did so, therefore all participants are included in reported analyses.
Within 72 hours of completing the baseline survey, participants in the experimental group were emailed an invitation to download Nod. Those in the control group were notified via email that they would receive access to the app after four weeks, and advised to await a download invitation. Participants accessed Nod through their university single sign-on, thus preventing an individual from making multiple accounts.
Online assessments through Qualtrics Software (Salt Lake City, UT) were administered at baseline and at weeks 2, 4, and 8.

14a-i) Indicate if critical "secular events" fell into the study period

Indicate if critical "secular events" fell into the study period, e.g., significant changes in Internet resources available or "changes in computer hardware or Internet delivery resources"

	1	2	3	4	5	
subitem not at all important	○	○	○	○	○	essential

Does your paper address subitem 14a-i?

Copy and paste relevant sections from the manuscript (include quotes in quotation marks "like this" to indicate direct quotes from your manuscript), or elaborate on this item by providing additional information not in the ms, or briefly explain why the item is not applicable/relevant for your study

Your answer

14b) Why the trial ended or was stopped (early)

Does your paper address CONSORT subitem 14b? *

Copy and paste relevant sections from the manuscript (include quotes in quotation marks "like this" to indicate direct quotes from your manuscript), or elaborate on this item by providing additional information not in the ms, or briefly explain why the item is not applicable/relevant for your study

N/A

15) A table showing baseline demographic and clinical characteristics for each group

NPT: When applicable, a description of care providers (case volume, qualification, expertise, etc.) and centers (volume) in each group

Does your paper address CONSORT subitem 15? *

Copy and paste relevant sections from the manuscript (include quotes in quotation marks "like this" to indicate direct quotes from your manuscript), or elaborate on this item by providing additional information not in the ms, or briefly explain why the item is not applicable/relevant for your study

Table 1

15-i) Report demographics associated with digital divide issues

In ehealth trials it is particularly important to report demographics associated with digital divide issues, such as age, education, gender, social-economic status, computer/Internet/ehealth literacy of the participants, if known.

	1	2	3	4	5	
subitem not at all important	○	○	○	○	○	essential

Does your paper address subitem 15-i? *

Copy and paste relevant sections from the manuscript (include quotes in quotation marks "like this" to indicate direct quotes from your manuscript), or elaborate on this item by providing additional information not in the ms, or briefly explain why the item is not applicable/relevant for your study

Table 1

16) For each group, number of participants (denominator) included in each analysis and whether the analysis was by original assigned groups

16-i) Report multiple “denominators” and provide definitions

Report multiple “denominators” and provide definitions: Report N’s (and effect sizes) “across a range of study participation [and use] thresholds” [1], e.g., N exposed, N consented, N used more than x times, N used more than y weeks, N participants “used” the intervention/comparator at specific pre-defined time points of interest (in absolute and relative numbers per group). Always clearly define “use” of the intervention.

	1	2	3	4	5	
subitem not at all important	○	○	○	○	○	essential

Does your paper address subitem 16-i? *

Copy and paste relevant sections from the manuscript (include quotes in quotation marks "like this" to indicate direct quotes from your manuscript), or elaborate on this item by providing additional information not in the ms, or briefly explain why the item is not applicable/relevant for your study

N/A - intent-to-treat analysis

16-ii) Primary analysis should be intent-to-treat

Primary analysis should be intent-to-treat, secondary analyses could include comparing only “users”, with the appropriate caveats that this is no longer a randomized sample (see 18-i).

	1	2	3	4	5	
subitem not at all important	○	○	○	○	○	essential

Does your paper address subitem 16-ii?

Copy and paste relevant sections from the manuscript (include quotes in quotation marks "like this" to indicate direct quotes from your manuscript), or elaborate on this item by providing additional information not in the ms, or briefly explain why the item is not applicable/relevant for your study

"Analyses of all outcome variables utilized an ‘intent-to-treat approach’, which included all available data from participants randomly assigned to the experimental and control groups.
"

17a) For each primary and secondary outcome, results for each group, and the estimated effect size and its precision (such as 95% confidence interval)

Does your paper address CONSORT subitem 17a? *

Copy and paste relevant sections from the manuscript (include quotes in quotation marks "like this" to indicate direct quotes from your manuscript), or elaborate on this item by providing additional information not in the ms, or briefly explain why the item is not applicable/relevant for your study

Loneliness
Descriptive examination of means revealed that both groups' loneliness scores declined slightly from baseline to week 4 (Table 3). Step 1 of the analyses, which examined condition differences in loneliness at week 4 controlling for baseline scores, showed no evidence for an overall effect of treatment on loneliness ($F_{1,211}=0.05$, $P=.82$; $\eta p2 = <.001$).
Step 2 of the analyses revealed a significant condition x baseline depression interaction ($F_{1,209}=9.65$, $P=.002$; $\eta p2 = .04$). To interpret this interaction, we conducted follow-up analyses of the simple slopes of baseline depression on week 4 loneliness for each condition separately. These analyses indicated that within the control group, there was a significant positive relationship between baseline depression and week 4 loneliness. In contrast, there was no significant relationship between baseline depression and week 4 loneliness within the experimental group (Table 4), suggesting that Nod buffered participants high in baseline depression from experiencing heightened mid-quarter loneliness (Figure 2).

Mental Health Indicators
Analyses in Step 1 showed no evidence for an overall effect of treatment on any of the four indices of mental health (ie, week 4 depression, anxiety, social anxiety, or sleep quality), all Fs <1.60 , Ps >.20. Step 2 of the analyses revealed a significant condition x baseline loneliness interaction to predict week 4 depression ($F_{1,209}=5.17$, $P=.02$; $\eta p2 = .02$) and week 4 sleep quality $F_{1,208}=8.26$, $P=.004$ $\eta p2 = .04$). Similar to the pattern observed for week 4 loneliness, simple slope analyses indicated that Nod buffered participants with higher baseline loneliness against heightened mid-quarter depression and poor sleep quality (Table 4; Figure 2). Baseline loneliness did not significantly moderate the effect of condition on week 4 anxiety or social anxiety (both Fs <1.80 , Ps >.18).

College Adjustment Indicators
There was no evidence for an overall effect of treatment on any of the three continuous indices of college adjustment (ie, week 4 social support, campus belonging, or social adjustment to college), all Fs <1.40, Ps>.23. However, the experimental group was more likely to report that they definitely intended to return to campus in the upcoming school year than the control (OR=2.11; 95% CI [1.00-4.49], z=1.95, P=.051).
Step 2 of the analyses revealed a significant condition x baseline loneliness interaction to predict week 4 social support ($F_{1,210}=4.05$, $P=.045$; $\eta p2 = .02$), and campus belonging ($F_{1,209}=9.44$, $P=.002$; $\eta p2 = .04$). The condition x baseline loneliness interaction to predict week 4 social adjustment to college approached but did not reach statistical significance ($F_{1,210}=3.66$, $P=.06$; $\eta p2 = .02$). Simple slope analyses suggested that Nod buffered participants with higher baseline loneliness against reduced social support, campus belonging, and social adjustment at week 4 (Table 4; Figure 2).
Additionally, the significant main effect of condition on intention to return was moderated by a condition x baseline loneliness interaction (OR=1.29; 95% CI [1.09-1.54]; z=2.90, P=.004). Probing of this interaction revealed that within the control group, the odds of "definitely" intending to return to campus significantly decreased as baseline loneliness increased. In contrast, in the experimental group the odds of intending to return significantly increased as baseline loneliness increased (Table 4).

17a-i) Presentation of process outcomes such as metrics of use and intensity of use

In addition to primary/secondary (clinical) outcomes, the presentation of process outcomes such as metrics of use and intensity of use (dose, exposure) and their operational definitions is critical. This does not only refer to metrics of attrition (13-b) (often a binary variable), but also to more continuous exposure metrics such as “average session length”. These must be accompanied by a technical description how a metric like a “session” is defined (e.g., timeout after idle time) [1] (report under item 6a).

	1	2	3	4	5	
subitem not at all important	○	○	○	○	○	essential

Does your paper address subitem 17a-i?

Copy and paste relevant sections from the manuscript (include quotes in quotation marks "like this" to indicate direct quotes from your manuscript), or elaborate on this item by providing additional information not in the ms, or briefly explain why the item is not applicable/relevant for your study

Your answer

17b) For binary outcomes, presentation of both absolute and relative effect sizes is recommended

Does your paper address CONSORT subitem 17b? *

Copy and paste relevant sections from the manuscript (include quotes in quotation marks "like this" to indicate direct quotes from your manuscript), or elaborate on this item by providing additional information not in the ms, or briefly explain why the item is not applicable/relevant for your study

N/A

18) Results of any other analyses performed, including subgroup analyses and adjusted analyses, distinguishing pre-specified from exploratory

Does your paper address CONSORT subitem 18? *

Copy and paste relevant sections from the manuscript (include quotes in quotation marks "like this" to indicate direct quotes from your manuscript), or elaborate on this item by providing additional information not in the ms, or briefly explain why the item is not applicable/relevant for your study

N/A

18-i) Subgroup analysis of comparing only users

A subgroup analysis of comparing only users is not uncommon in ehealth trials, but if done, it must be stressed that this is a self-selected sample and no longer an unbiased sample from a randomized trial (see 16-iii).

	1	2	3	4	5	
subitem not at all important	○	○	○	○	○	essential

Does your paper address subitem 18-i?

Copy and paste relevant sections from the manuscript (include quotes in quotation marks "like this" to indicate direct quotes from your manuscript), or elaborate on this item by providing additional information not in the ms, or briefly explain why the item is not applicable/relevant for your study

Your answer

19) All important harms or unintended effects in each group

(for specific guidance see CONSORT for harms)

Does your paper address CONSORT subitem 19? *

Copy and paste relevant sections from the manuscript (include quotes in quotation marks "like this" to indicate direct quotes from your manuscript), or elaborate on this item by providing additional information not in the ms, or briefly explain why the item is not applicable/relevant for your study

N/A no harms

19-i) Include privacy breaches, technical problems

Include privacy breaches, technical problems. This does not only include physical “harm” to participants, but also incidents such as perceived or real privacy breaches [1], technical problems, and other unexpected/unintended incidents. “Unintended effects” also includes unintended positive effects [2].

	1	2	3	4	5	
subitem not at all important	○	○	○	○	○	essential

Does your paper address subitem 19-i?

Copy and paste relevant sections from the manuscript (include quotes in quotation marks "like this" to indicate direct quotes from your manuscript), or elaborate on this item by providing additional information not in the ms, or briefly explain why the item is not applicable/relevant for your study

Your answer

19-ii) Include qualitative feedback from participants or observations from staff/researchers

Include qualitative feedback from participants or observations from staff/researchers, if available, on strengths and shortcomings of the application, especially if they point to unintended/unexpected effects or uses. This includes (if available) reasons for why people did or did not use the application as intended by the developers.

	1	2	3	4	5	
subitem not at all important	○	○	○	○	○	essential

Does your paper address subitem 19-ii?

Copy and paste relevant sections from the manuscript (include quotes in quotation marks "like this" to indicate direct quotes from your manuscript), or elaborate on this item by providing additional information not in the ms, or briefly explain why the item is not applicable/relevant for your study

Your answer

DISCUSSION

22) Interpretation consistent with results, balancing benefits and harms, and considering other relevant evidence

NPT: In addition, take into account the choice of the comparator, lack of or partial blinding, and unequal expertise of care providers or centers in each group

22-i) Restate study questions and summarize the answers suggested by the data, starting with primary outcomes and process outcomes (use)

Restate study questions and summarize the answers suggested by the data, starting with primary outcomes and process outcomes (use).

	1	2	3	4	5	
subitem not at all important	○	○	○	○	○	essential

Does your paper address subitem 22-i? *

Copy and paste relevant sections from the manuscript (include quotes in quotation marks "like this" to indicate direct quotes from your manuscript), or elaborate on this item by providing additional information not in the ms, or briefly explain why the item is not applicable/relevant for your study

In a 4 week pilot randomized trial, Nod had specific benefits for students who entered college with elevated risk (ie, heightened loneliness and depression) relative to their peers. Exposure to Nod buffered vulnerable first-year college students from experiencing heightened mid-semester loneliness and depressive symptoms, and protected against poor sleep quality, reduced social support, and reduced campus belonging. Notably, the experimental group was more likely to report that they would definitely return to campus in the upcoming school year, a benefit that was particularly pronounced for vulnerable students, who are at heightened risk of early attrition.[21] Results support using app-based interventions to facilitate social connection, especially among first-year students experiencing elevated loneliness or depressive symptoms during key moments of social transition.
Less vulnerable students (i.e. those with average to low levels of baseline loneliness and depression) did not derive significant benefits from Nod. These students may have had less need for the provided skills, and thus may have used Nod less frequently and benefited from it less. This possibility is supported by exploratory analyses demonstrating that baseline loneliness was positively associated with all measures of app engagement in the experimental group (all rs > .27, Ps<.05), indicating that less vulnerable students used Nod less frequently.

22-ii) Highlight unanswered new questions, suggest future research

Highlight unanswered new questions, suggest future research.

	1	2	3	4	5	
subitem not at all important	○	○	○	○	○	essential

Does your paper address subitem 22-ii?

Copy and paste relevant sections from the manuscript (include quotes in quotation marks "like this" to indicate direct quotes from your manuscript), or elaborate on this item by providing additional information not in the ms, or briefly explain why the item is not applicable/relevant for your study

The randomized design of this trial extends the promising findings of similar interventions for college students[38], and bolsters confidence that loneliness can be addressed digitally. Future work will aim to improve upon app engagement, and to address loneliness during other key social transitions, and among other young populations who may benefit from digital interventions to support social connectedness.

20) Trial limitations, addressing sources of potential bias, imprecision, and, if relevant, multiplicity of analyses

20-i) Typical limitations in ehealth trials

Typical limitations in ehealth trials: Participants in ehealth trials are rarely blinded. Ehealth trials often look at a multiplicity of outcomes, increasing risk for a Type I error. Discuss biases due to non-use of the intervention/usability issues, biases through informed consent procedures, unexpected events.

	1	2	3	4	5	
subitem not at all important	○	○	○	○	○	essential

Does your paper address subitem 20-i? *

Copy and paste relevant sections from the manuscript (include quotes in quotation marks "like this" to indicate direct quotes from your manuscript), or elaborate on this item by providing additional information not in the ms, or briefly explain why the item is not applicable/relevant for your study

Several limitations motivate further investigation. First, as an initial pilot trial, our sample size was relatively small and the study duration relatively short. Future larger trials should replicate the observed effects, and examine the psychological mechanisms through which Nod buffers vulnerable students (eg, by promoting social growth mindsets, social risk taking, or self-compassion). Second, on average, participants did not engage extensively with Nod, impeding meaningful analyses of the relationship between engagement and outcomes. While we observed weak positive associations between engagement and outcomes, future studies with wider ranges of engagement are needed to better clarify findings. Third, similar to other tech-based behavioral interventions, it was not possible to blind participants to condition. Finally, although the sample was racially and socioeconomically diverse, more women than men expressed interest in participating, and were included in the final sample. Participants were also motivated to participate in a trial and were incentivized to download Nod, potentially weakening the generalizability of the findings. Naturalistic study of engagement outside of a clinical trial could provide more generalizable insights regarding Nod’s benefits.

21) Generalisability (external validity, applicability) of the trial findings

NPT: External validity of the trial findings according to the intervention, comparators, patients, and care providers or centers involved in the trial

21-i) Generalizability to other populations

Generalizability to other populations: In particular, discuss generalizability to a general Internet population, outside of a RCT setting, and general patient population, including applicability of the study results for other organizations

	1	2	3	4	5	
subitem not at all important	○	○	○	○	○	essential

Does your paper address subitem 21-i?

Copy and paste relevant sections from the manuscript (include quotes in quotation marks "like this" to indicate direct quotes from your manuscript), or elaborate on this item by providing additional information not in the ms, or briefly explain why the item is not applicable/relevant for your study

Finally, although the sample was racially and socioeconomically diverse, more women than men expressed interest in participating, and were included in the final sample. Participants were also motivated to participate in a trial and were incentivized to download Nod, potentially weakening the generalizability of the findings. Naturalistic study of engagement outside of a clinical trial could provide more generalizable insights regarding Nod's benefits.

21-ii) Discuss if there were elements in the RCT that would be different in a routine application setting

Discuss if there were elements in the RCT that would be different in a routine application setting (e.g., prompts/reminders, more human involvement, training sessions or other co-interventions) and what impact the omission of these elements could have on use, adoption, or outcomes if the intervention is applied outside of a RCT setting.

	1	2	3	4	5	
subitem not at all important	○	○	○	○	○	essential

Does your paper address subitem 21-ii?

Copy and paste relevant sections from the manuscript (include quotes in quotation marks "like this" to indicate direct quotes from your manuscript), or elaborate on this item by providing additional information not in the ms, or briefly explain why the item is not applicable/relevant for your study

N/A - none

OTHER INFORMATION

23) Registration number and name of trial registry

Does your paper address CONSORT subitem 23? *

Copy and paste relevant sections from the manuscript (include quotes in quotation marks "like this" to indicate direct quotes from your manuscript), or elaborate on this item by providing additional information not in the ms, or briefly explain why the item is not applicable/relevant for your study

ClinicalTrials.gov Identifier: NCT04164654

24) Where the full trial protocol can be accessed, if available

Does your paper address CONSORT subitem 24? *

Cite a Multimedia Appendix, other reference, or copy and paste relevant sections from the manuscript (include quotes in quotation marks "like this" to indicate direct quotes from your manuscript), or elaborate on this item by providing additional information not in the ms, or briefly explain why the item is not applicable/relevant for your study

ClinicalTrials.gov Identifier: NCT04164654

25) Sources of funding and other support (such as supply of drugs), role of funders

Does your paper address CONSORT subitem 25? *

Copy and paste relevant sections from the manuscript (include quotes in quotation marks "like this" to indicate direct quotes from your manuscript), or elaborate on this item by providing additional information not in the ms, or briefly explain why the item is not applicable/relevant for your study

Nod is a mobile app co-developed by Grit Digital Health and Hopelab. While both organizations own rights to the Nod product, Grit Digital Health is solely responsible for the commercial operation and distribution of Nod. As co-owner, Hopelab receives a limited portion of Nod sale net proceeds. The research reported here as well as the development of Nod was supported by the nonprofit Hopelab Foundation. Hopelab develops behavioral interventions to improve the health and well-being of young people. The design, conduct, analysis, and reporting of this study represents a scientific collaboration between Hopelab, Jennifer Pfeifer, and Brittany Davis at the University of Oregon, and Kevin Delucchi at the University of California, San Francisco and Weill Institute for Neurosciences. Emma Bruehlman-Senecal, Cayce Hook, Caroline Fitzgerald, Jana Haritatos, and Danielle Ramo are employed by Hopelab Foundation. The study sponsor was involved in the study design, collection, analysis, and interpretation of data, writing of the article, and decision to submit it for publication.

X27) Conflicts of Interest (not a CONSORT item)

X27-i) State the relation of the study team towards the system being evaluated

In addition to the usual declaration of interests (financial or otherwise), also state the relation of the study team towards the system being evaluated, i.e., state if the authors/evaluators are distinct from or identical with the developers/sponsors of the intervention.

	1	2	3	4	5	
subitem not at all important	○	○	○	○	○	essential

Does your paper address subitem X27-i?

Copy and paste relevant sections from the manuscript (include quotes in quotation marks "like this" to indicate direct quotes from your manuscript), or elaborate on this item by providing additional information not in the ms, or briefly explain why the item is not applicable/relevant for your study

Your answer

About the CONSORT EHEALTH checklist

As a result of using this checklist, did you make changes in your manuscript? *

- ○ yes, major changes
- ◉ yes, minor changes
- ○ no

What were the most important changes you made as a result of using this checklist?

Your answer

How much time did you spend on going through the checklist INCLUDING making changes in your manuscript *

3 hours

As a result of using this checklist, do you think your manuscript has improved? *

- ○ yes
- ◉ no
- ○ Other:

Would you like to become involved in the CONSORT EHEALTH group?

This would involve for example becoming involved in participating in a workshop and writing an "Explanation and Elaboration" document

- ○ yes
- ◉ no
- ○ Other:

Any other comments or questions on CONSORT EHEALTH

Suggest switching to another format that allows saving progress, rather than Google Forms

STOP - Save this form as PDF before you click submit

To generate a record that you filled in this form, we recommend to generate a PDF of this page (on a Mac, simply select "print" and then select "print as PDF") before you submit it.

When you submit your (revised) paper to JMIR, please upload the PDF as supplementary file.

Don't worry if some text in the textboxes is cut off, as we still have the complete information in our database. Thank you!

Final step: Click submit !

Click submit so we have your answers in our database!

Submit

Never submit passwords through Google Forms.

This form was created outside of your domain. Report Abuse - Terms of Service - Privacy Policy

Google Forms
